# A synergetic effect of BARD1 mutations on tumorigenesis

**DOI:** 10.1038/s41467-021-21519-3

**Published:** 2021-02-23

**Authors:** Wenjing Li, Xiaoyang Gu, Chunhong Liu, Yanyan Shi, Pan Wang, Na Zhang, Rui Wu, Liang Leng, Bingteng Xie, Chen Song, Mo Li

**Affiliations:** 1 Center for Reproductive Medicine, Department of Obstetrics and Gynecology, Peking University Third Hospital, Beijing, China; 2 National Clinical Research Center for Obstetrics and Gynecology, Peking University Third Hospital, Beijing, China; 3 Beijing Key Laboratory of Reproductive Endocrinology and Assisted Reproductive Technology (Peking University Third Hospital), Beijing, China; 4 Center for Quantitative Biology, Academy for Advanced Interdisciplinary Studies, Peking University, Beijing, China; 5 Research Center of Clinical Epidemiology, Peking University Third Hospital, Beijing, China; 6 Department of Pathology, Peking University Third Hospital, School of Basic Medical Sciences, Peking University Health Science Center, Beijing, China; 7 Key Laboratory of Beijing for Identification and Safety Evaluation of Chinese Medicine, China Academy of Chinese Medical Sciences, Institute of Chinese Materia Medica, Beijing, China; 8 Peking-Tsinghua Center for Life Sciences, Academy for Advanced Interdisciplinary Studies, Peking University, Beijing, China

**Keywords:** Breast cancer, DNA damage response

## Abstract

To date, a large number of mutations have been screened from breast and ovarian cancer patients. However, most of them are classified into benign or unidentified alterations due to their undetectable phenotypes. Whether and how they could cause tumors remains unknown, and this significantly limits diagnosis and therapy. Here, in a study of a family with hereditary breast and ovarian cancer, we find that two BARD1 mutations, P24S and R378S, simultaneously exist in cis in surviving cancer patients. Neither of the single mutations causes a functional change, but together they synergetically impair the DNA damage response and lead to tumors in vitro and in vivo. Thus, our report not only demonstrates that BARD1 defects account for tumorigenesis but also uncovers the potential risk of synergetic effects between the large number of cis mutations in individual genes in the human genome.

## Abstract (web summary)

Mutations in the BARD1 gene have been identified as risk factors in breast and ovarian cancers. Here the authors reveal a synergetic effect between the occurance of two cis mutations in the BARD1 gene affecting the cellular DNA damage response and consequently tumorigenesis.

## Introduction

It has been estimated that in each cell in our bodies up to 10^5^ DNA lesions are induced every day by cellular metabolism, spontaneous chemical reactions, and exogenous physical agents^1^. To contend with these threats, cells have evolved the DNA damage response (DDR) system to sense and repair lesions^2,3^. Failure of the DNA repair system causes genome instability resulting from mutations such as chromosomal rearrangements and deletions, which potentially leads to tumorigenesis^4,5^. Thus, accurate regulation of DDR is essential for genome integrity and cancer prevention. As a core DDR factor, BRCA1 mediates homologous recombination (HR) repair of DNA double-strand breaks (DSBs), the most deleterious type of DNA lesion^3,6,7^. It is well known that BRCA1 dysfunction is highly associated with breast and ovarian tumorigenesis^8–10^. In women, germline mutations of *BRCA1* account for up to a 90% risk of developing breast cancer and ~40% risk of developing ovarian cancer^11,12^. However, mutations in BRCA1 per se only explain a part of familial breast and ovarian cancers. Identification of cancer-causing mutations in BRCA1-associated genes in clinical patients is urgently needed for developing early diagnosis methods and treatments.

In cells, BRCA1 and BRCA1-associated RING-domain protein 1 (BARD1) interact via their N-terminal RING domains to form a heterodimeric complex^13,14^. This interaction stabilizes both BRCA1 and BARD1 because the respective monomers are unstable^15,16^. Mounting evidence suggests that the BRCA1/BARD1 complex has important roles in the DDR system. Upon genotoxic stress, BARD1 serves as a BRCA1 nuclear chaperone that promotes the formation and retention of BRCA1 foci, and these foci are colocalized with DNA repair effectors such as BRCA2 and RAD51^17–20^. Biochemical analyses have revealed that the individual BRCA1 and BARD1 proteins have very low ubiquitin ligase activity, whereas the complex works as an efficient E3 ligase^21,22^. The intimate binding between the two proteins ensures an early response at the DNA damage site^7^, promotes DNA resection on the damaged chromatin^23^, and enhances the recombinase activity of RAD51 by facilitating the assembly of an intermediate of DNA joint formation^24^. Interestingly, *Brca1* and *Bard1* knockout mice are both embryonic lethal with striking similarities in genomic instability resulting from the accumulation of DNA lesions^25,26^, meaning that BARD1 is as important as BRCA1 for cell viability.

To date, numerous human BARD1 mutations have been screened from large-scale sequencing of clinical patients. However, almost all of these mutations are classified as benign or unidentified mutations^27,28^, although several of them are suspected to pose a potential risk for breast and ovarian cancers^29,30^. In particular, integrated evidence of clinical mutations combined with molecular functions is still absent. Here, in a study of a hereditary breast and ovarian cancer susceptible (HBOC) family, we find that two BARD1 mutations (P24S and R378S) simultaneously exist in surviving cancer patients. At the molecular and biochemical level, P24S weakens the affinity between BARD1 and BRCA1, whereas R378S attenuates nuclear location of the BRCA1/BARD1 complex. Each single BARD1 mutation causes no obvious cellular phenotype, but combines in cis resulting in impaired DDR and DNA lesions. Non-transformed cells bearing the double mutations show significant genome instability and shift to transformed cells after exposure to DNA damage agents in vitro and in vivo. Thus, our study not only identifies BARD1 mutations affecting the BRCA1/BARD1 complex but also uncovers the synergetic effect of cis mutations in individual genes on tumorigenesis, which updates the understanding of how non-effective mutations cause cancers.

## Results

### BARD1 mutations identified in an HBOC family

**Fig. 1:**
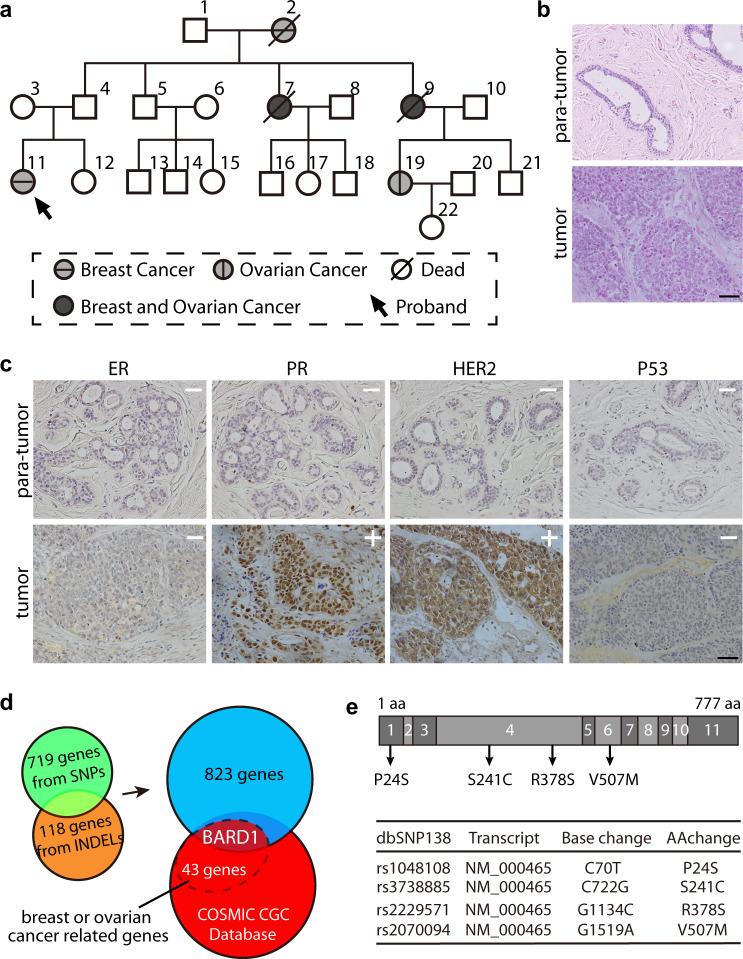
Mutational analysis of a hereditary breast and ovarian cancer (HBOC) family. **a** Pedigree of the HBOC family. Circles indicate females and squares indicate males. The black arrow indicates the proband. Genomic DNA samples of the proband (#11) and all surviving female siblings (#12, 15, 17, 19) (except #22, a 9-year-old girl) were analyzed with Whole Genome Sequencing. **b** H&E staining of proband mammary tissues. Mammary tissues from proband para-tumor and tumor mass were harvested and H&E stained. Scale bar, 50 μm. **c** Pathological marker staining of proband mammary tissues. Mammary tissues from proband para-tumor and tumor mass were harvested and stained for ER, PR, HER2, and P53. Signal positive and negative are denoted by ‘+’ and ‘−’, respectively. Scale bar, 50 μm. **d** Gene screening flowchart by bioinformatics analysis. Different clusters were generated from the WGS data via multiple bioinformatic approaches (SIFT, PolyPhen2, PROVEAN, etc) combined with public database (COSMIC CGC database) searching. Only the *BARD1* gene was filtered out as a candidate pathogenic gene highly related to both breast and ovarian cancer. **e** *BARD1* mutations in the HBOC family. The *BARD1* mutations are annotated across the coding sequence of the gene’s 11 exons. The SNP IDs and corresponding amino-acid changes in *BARD1* are summarized.

In an HBOC family (Fig. 1a), a 38-year-old proband (Person-11) was diagnosed with invasive ductal carcinoma breast cancer in our hospital. Hematoxylin and eosin (H&E) staining of biopsied tissue revealed cancerous cells in the tumor mass, and normal mammary morphology in the para-tumor tissue (Fig. 1b). In terms of pathological markers, the tumor mass was estrogen receptor (ER) negative, progesterone receptor (PR) positive, and human epidermal growth factor receptor 2 (HER2) positive, with low expression of P53 (Fig. 1c). Among other members of the family, person-2 was affected with invasive ductal breast cancer, person-19 was affected with bilateral high-grade serous ovarian carcinoma, while person-7 and person-9 suffered from both breast and ovarian cancers. Detailed information about the family is included in Supplementary Data 1. Collectively, these findings clearly suggest that this family has a characteristic^31^ HBOC pedigree.

To identify cancer-associated genes in the HBOC family, we sampled the peripheral blood of all surviving female members including person−11, −12, −15, −17, and −19 for whole-genomic sequencing (WGS). Person-22 was not included, as she is a 9-year-old girl. We deep sequenced each sample to a coverage depth of ~29X (~88 GB raw data/sample, Supplementary Data 2), and then detected single nucleotide polymorphisms (SNPs), insertion and deletion (INDEL) mutations, structural variations (SVs), copy number variations (CNVs). Analysis of these mutations revealed that the family had mutation loads within normal ranges (e.g., ~2.8 million SNPs were found in each family member, as well as 0.5 million INDELs, 6500 SVs, and 1300 CNVs). These variants were further annotated using various functional and pathogenic databases such as SIFT, PolyPhen2, and PROVEAN^32–34^. None of the detected SVs or CNVs have been previously associated with breast or ovarian cancer. Of note, 792 potential pathogenic SNPs were found (in 719 genes) (Supplementary Data 3), whereas 125 INDELs were present in 118 genes (Supplementary Data 4). To narrow down the candidate genes, we employed COSMIC Cancer Gene Census, a database curating comprehensive information about the genes driving human cancers^35,36^. Interestingly, this analysis identified only *BARD1* as being potentially associated with breast and ovarian cancers (Fig. 1d). The HBOC family polymorphisms at the *BARD1* locus (rs1048108, rs3738885, rs2229571, and rs2070094, further confirmed by Sanger sequencing) are predicted to result in four amino-acid substitutions: P24S, S241C, R378S, and V507M, respectively (Fig. 1e).

### BARD1 double mutations of P24S and R378S jointly impair DDR

**Fig. 2:**
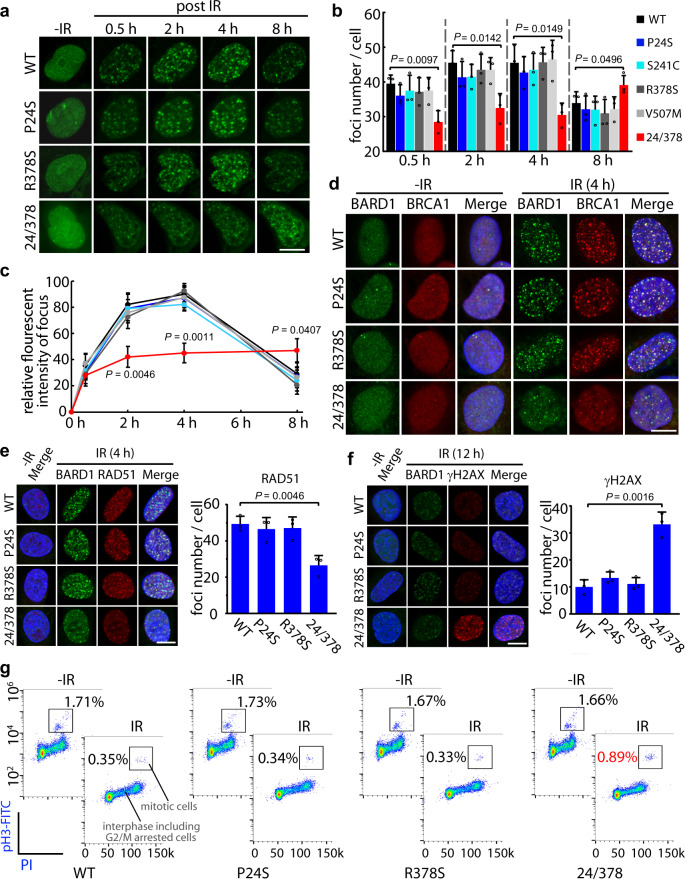
Effect of BARD1 mutations on DNA damage response. **a** Foci formation of BARD1 variants in living cells upon DNA damage. U2OS cells expressing GFP-BARD1^WT^ and the different BARD1 variants (GFP-BARD1^P24S^, BARD1^S241C^, BARD1^R378S^, BARD1^V507M^, or BARD1^P24S/R378S^) were treated with 5 Gy IR followed by live-cell imaging for 8 h. Scale bar, 10 μm. **b** Foci number in different BARD1 variant cells. The foci number in each cell expressing GFP-BARD1^WT^ and GFP-BARD1 variants were counted at the indicated time points after IR treatment. At least 30 cells were included for each group. Three biologically independent replicates were performed. Data are presented as mean values ± SD. *P* values are calculated by unpaired two-tailed Student’s *t* tests. **c** Individual foci dynamics of BARD1 variants in living cells. The fluorescence intensity of GFP-BARD1^WT^ and GFP-BARD1 variants were measured at the indicated time points. Twenty foci per cell from at least ten cells in each group were analyzed. Three biologically independent replicates were performed. Data are presented as mean values ± SD. *P* values are calculated by unpaired two-tailed Student’s *t* tests. **d** Immunofluorescence of endogenous BRCA1 in BARD1 mutant cells upon DNA damage. Cells expressing GFP-BARD1^WT^ or GFP-BARD1 variants were treated with 5 Gy IR followed by the staining of endogenous BRCA1. Scale bar, 10 μm. **e** Cells expressing GFP-BARD1^WT^ or GFP-BARD1 variants were treated with 5 Gy IR followed by the staining of endogenous RAD51. Scale bar, 10 μm. The RAD51 foci number in each cell was counted. At least 30 cells were included for each group. Three biologically independent replicates were performed. Data are presented as mean values ± SD. *P* values are calculated by unpaired two-tailed Student’s *t* tests. **f** Cells expressing GFP-BARD1^WT^ or GFP-BARD1 variants were treated with 5 Gy IR followed by a recovery of 12 h and stained for endogenous γH2AX. Scale bar, 10 μm. The γH2AX foci number in each cell was counted. At least 30 cells were included for each group. Three biologically independent replicates were performed. Data are presented as mean values ± SD. *P* values are calculated by unpaired two-tailed Student’s *t* tests. **g** G2/M checkpoint activation of cells expressing different BARD1 variants. Cells were treated with or without IR followed by phospho-histone 3 staining. Cells were examined by flow cytometry and the phospho-histone 3-positive population (i.e., mitotic cells) are boxed. Source data are provided as a Source Data file.

To determine the potential effect of these BARD1 mutations on tumorigenesis, we tested the response of each single BARR1 mutant variant to DNA damage with in vitro ionizing radiation (IR) damage assays. U2OS cells with knockout of endogenous BARD1 generated using the CRISPR/Cas9 system (Supplementary Fig. 1a) and stably expressing the wild-type (WT) GFP-BARD1 fusion protein, or one of the four different HBOC family BARD1 mutant variants (P24S, S241C, R378S, and V507M, also expressed as GFP fusions) were treated with DNA-damaging IR, followed by BARD1 foci detection via live-cell imaging. No significant difference in foci number was found between WT BARD1 and the single-mutant variant. The response kinetics for the formation of individual foci by the single BARD1 mutant variants were also similar to those for the WT counterpart (Fig. 2a–c, Supplementary Fig. 1b). Thus, we were curious whether BARD1 protein variants bearing two mutations could potentially increase the risk of tumorigenesis. Looking back at the distribution of the BARD1 mutations in the HBOC family, we found that both of the surviving cancer patients shared alleles coding for two identical mutations: P24S and R378S. Using the cDNA libraries from the two patients, the full-length coding sequence of *BARD1* was amplified (Supplementary Fig. 1c). As each single *BARD1-*coding sequence was derived from a single allele from Patient-11 or −19, we sequenced the coding sequences from single colonies (10 clones were randomly picked for each patient) for distinguishing whether the two mutations were linked to the same allele. As seen in Supplementary Fig. 1d, the double mutations of P24S and R378S co-existed in 3 out of 10 monoclones from Patient-11, whereas the other seven monoclones bore neither the P24S nor R378S mutation. In Patient-19, the double mutations co-existed in five monoclones and the other five monoclones did not contain these mutations (Supplementary Fig. 1d). All 20 monoclones exhibited an “all-or-none” pattern for the mutations, meaning that the two mutations are linked on the same allele. Combined with the sequencing data, we also calculated the possible LOD score (3.0103, indicated significant^37,38^) for the association of the BARD1-P24S/R378S variant with tumor occurrence within the HBOC pedigree. In addition, we tested the occurrence of loss-of-heterozygosity within the tumors. In all, 100 single tumor cells were harvested from the tumor masses of each patient followed by single-cell whole-genome amplification. In total, 26 out of 100 cells in the tumor of patient-11 (breast cancer) showed loss-of-heterozygosity (homozygous sequence for P24S). While 19 out of 100 cells in the tumor of patient-19 (ovarian cancer) showed loss-of-heterozygosity (Supplementary Fig. 1e). These data suggest that a certain degree of loss-of-heterozygosity occurred within the tumors of the patients. Next, we investigated the DDR of cells stably expressing the double-mutant BARD1 protein with the P24S/R378S substitutions. Interestingly, significantly attenuated formation of foci post-DNA damage was observed in these cells. In particular, the BARD1^P24S/R378S^ variant formed fewer foci during the early DDR (2 and 4 h post IR) accompanied by sluggish response compared with WT BARD1 (Fig. 2a–c).

Furthermore, we examined whether the double-mutant BARD1^P24S/R378S^ could affect the response of endogenous BRCA1 under DNA stress. No difference in BRCA1 foci formation was detected between cells expressing WT or single-mutant BARD1. However, cells expressing BARD1^P24S/R378S^ showed fewer and irregular BRCA1 foci after IR treatment (Fig. 2d, Supplementary Fig. 1f, g). It is known that BRCA1/BARD1 complex promotes HR repair^24^. We thus detected the foci formation of RAD51, a core recombinase for HR repair, in these IR-treated cells. No difference of RAD51 foci formation was observed between the single-mutant and WT cells. However, the BARD1^P24S/R378S^ cells showed reduced foci number of RAD51 (Fig. 2e). Accordingly, after recovery from IR treatment, the WT and single-mutant cells repaired most of DNA DSBs. While obvious DNA damages labeled by γH2AX still existed in the P24S/R378S double-mutant cells (Fig. 2f). These results indicate that the impaired DDR caused by the P24S/R378S double mutations weakens the ability of HR repair that leads to heavier accumulation of DNA damage.

It is well known that the BRCA1/BARD1 complex has potent ubiquitin ligase activity and is involved in multiple biological processes^6,21,22^. We thus investigated whether the double mutations affect the activity. The N-terminal fragment of BRCA1 (1–300 amino acids), which exhibits ubiquitin ligase activity^39,40^, and full length of WT and mutant BARD1 proteins were respectively expressed and purified (Supplementary Fig. 1h). An in vitro reaction was set up containing ubiquitin-activating enzyme (E1), ubiquitin-conjugating enzyme (E2), and the BRCA1-N and BARD1 peptides according to previous reports^23,41^. As shown in Supplementary Fig. 1i, ubiquitin-conjugation occurred in the reaction group with WT BRCA1/BARD1, as visualized by the formation of ubiquitin chains. The levels of ubiquitin-conjugation in the BARD1^S241C^, BARD1^R378S^, and BARD1^V507M^ groups were similar to that in WT control group. However, ~30% reduction in ubiquitin chains was observed in the BARD1^P24S^ and BARD1 ^P24S/R378S^ groups, indicating that the BARD1^P24S^ variant mildly weakens the ubiquitin ligase activity of the BRCA1/BARD1 complex. In parallel, we confirmed the in vitro results at the cellular level. U2OS cells (knockout of endogenous BARD1) stably expressing WT and different mutant BARD1 were treated with 5 Gy IR and lysed. The cell lysates were immunoprecipitated by BRCA1 antibody for sodium dodecyl sulfate (SDS) electrophoresis followed by western blot against ubiquitin antibody. The levels of ubiquitin-conjugation in cells expressing BARD1^S241C^, BARD1^R378S^, or BARD1^V507M^ showed no difference between those in cells expressing WT BARD1. However, the levels of ubiquitin-conjugation in BARD1^P24S^ and BARD1^P24S/R378S^-bearing cells were detectably lower (Supplementary Fig. 1j). These data indicate that BARD1^P24S^ mildly reduces the ubiquitin ligase activity of the BRCA1/BARD1 complex in vitro.

Following DSBs, cells are transiently arrested by G2/M checkpoint before mitosis that stops the proliferation of the damaged cells and allows their DNA repair^42^. Given accumulating evidence that the BRCA1/BARD1 complex participates in the regulation of DNA damage-induced G2/M checkpoint^18,43^, we monitored the mitotic cell population by examining phosphorylated histone H3, which is a standard assay for studying G2/M checkpoint^44,45^. As seen in Fig. 2g, after IR treatment, cells expressing the WT or single-mutant BARD1 were arrested before mitosis, as shown by a reduced number of mitotic cells detected by flow cytometry (e.g., 0.35% compared with 1.71%), which were marked by phospho-H3 positive staining. However, cells expressing BARD1^P24S/R378S^ showed a larger population of mitotic cells (0.89%) than observed for BARD1^WT^ cells, indicating a weakened G2/M checkpoint under DNA stress (Supplementary Fig. 1k). Taken together, these data suggest that BARD1^P24S/R378S^ impairs DDR and imply that the two mutations have a synergetic effect on cells.

### P24S mutation weakens the affinity between BARD1 and BRCA1

**Fig. 3:**
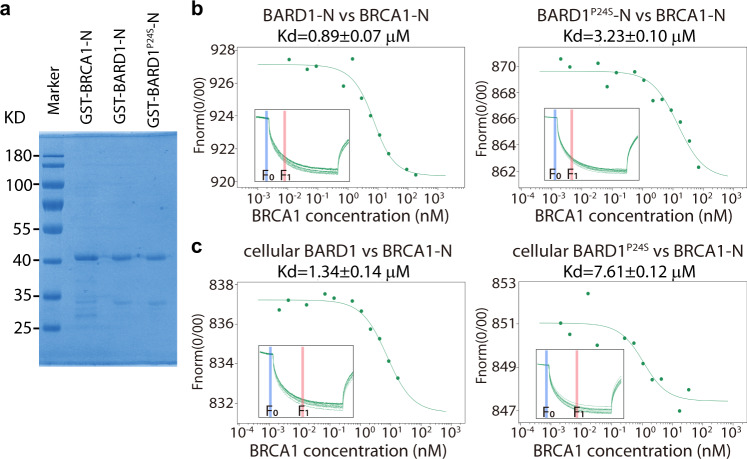
Binding affinity between BARD1^P24S^ and BRCA1. **a** Peptide expression and purification of recombinant N-terminals of BARD1 and BRCA1. The N-terminals of BRCA1 (1–147 aa), BARD1 (1–142 aa), and BARD1^P24S^ (1–142 aa) were expressed in *E. coli* with a GST tag followed by affinity purification. **b** In vitro binding between N-terminals of BARD1 and BRCA1. The binding affinity between BRCA1-N and BARD1-N or BARD1^P24S^-N was measured by microscale thermophoresis (MST). Inset, thermophoretic movement of fluorescently labeled proteins. *F*_norm_ = *F*_1_/*F*_0_ (*F*_norm_: normalized fluorescence; *F*_1_: fluorescence after thermodiffusion; *F*_0_: initial fluorescence or fluorescence after T-jump). *Kd*, dissociation constant. **c** Binding between BARD1 from cell lysates and recombinant BRCA1-N. U2OS cells expressing GFP-BARD1^WT^ or GFP-BARD1^P24S^ were lysed. The affinity between the fluorescent BARD1 and recombinant BRCA1-N was measured by MST. Inset, thermophoretic movement of fluorescent GFP-BARD1^WT^ or GFP-BARD1^P24S^.

To explore how the P24S and R378S mutations reciprocally enhance each other’s effects, we characterized their features individually. It is well known that BARD1 and BRCA1 form the stable heterodimer via their N-terminals^13,14^, we thus questioned whether the P24S mutation would affect the binding between BARD1 and BRCA1. As this single mutation did not cause observable phenotype on cells in our cellular assays, we performed a series of biochemical assays to quantify the affinity between BRCA1 and mutated BARD1. The N-terminals of BRCA1 (1–147 aa, BRCA1-N), BARD1 (1–142 aa, BARD1-N), and BARD1^P24S^ (BARD1^P24S^-N) fused with GST tag were expressed and purified (Fig. 3a). The direct binding between BRCA1-N and BARD1-N or BARD1^P24S^-N was measured by microscale thermophoresis (MST), a sensitive technology for affinity analysis of biomolecules^46,47^. Analysis of the binding curves showed that the dissociation constant (Kd) of BARD1-N and BRCA1-N was 0.89 μM; in contrast the Kd for BARD1^P24S^-N and BRCA1-N was significantly higher (3.23 μM), meaning a decreased binding affinity (Fig. 3b). A semi-in vivo assay was also performed in which the binding affinity between BRCA1-N and cellular BARD1 was tested. Purified GST-BRCA1-N was incubated with lysates of U2OS cells expressing GFP-BARD1^WT^ or GFP-BARD1^P24S^ followed by MST measurement. Consistent with in vitro data, BRCA1-N bound with stronger affinity to cellular GFP-BARD1^WT^ than to cellular GFP-BARD1^P24S^ (Fig. 3c).

To further investigate the effect of the P24S substitution on the BARD1-BRCA1 interaction, we performed molecular dynamics (MD) simulation to assess protein dynamics and atomic-level influences on binding affinity^48,49^. In the simulation, the root-mean-square deviation of the BARD1^P24S^/BRCA1 RING-domain heterodimer was larger than that of the WT heterodimer (Supplementary Fig. 2a), indicating that the P24S mutation may destabilize the heterodimer to some extent. The N-terminal residues near S24 of BARD1^P24S^ are likely to form fewer hydrogen bonds with BRCA1 (Supplementary Fig. 2b), leading to weaker binding between BRCA1 and BARD1^P24S^. Visualizing the simulation trajectories, we could see that the coil region around P24 of WT BARD1 forms favorable interactions with BRCA1 (Supplementary Fig. 2c), whereas the P24S mutation tends to form a hydrogen bond with D117 of BARD1 (Supplementary Fig. 2d), thus prohibiting interactions between the BARD1-N-terminal coil and BRCA1, likely resulting in a weaker overall binding. Collectively, our empirical data demonstrate that the BARD1 P24S substitution biochemically weakens BARD1’s affinity with BRCA1, and our simulations present a plausible chemical mechanism to explain this weakened interaction.

### R378-containing region partially contributes to nuclear localization of BARD1

**Fig. 4:**
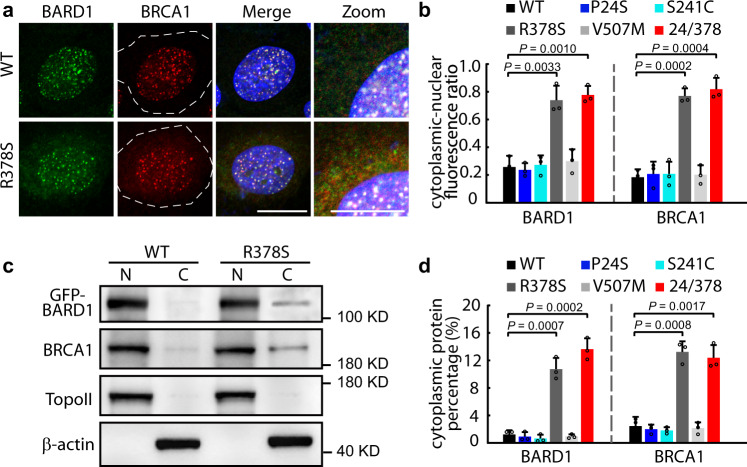
Cytoplasmic translocation of BARD1^R378S^. **a** Immunofluorescence of cytoplasmic BARD1 and BRCA1. Cells expressing GFP-BARD1^WT^ or GFP-BARD1 variants were treated with 5 Gy IR followed by immunostaining of endogenous BRCA1 4 h after IR. Whole cells are circled by dashed lines. Scale bar, 10 μm. **b** Cytoplasmic-nuclear fluorescence ratio of BARD1 and BRCA1. Fluorescence intensity of BARD1 and BRCA1 in the cytoplasm and the nucleus (note that foci were excluded from the nuclear intensity readings) were recorded. Ratios of cytoplasmic versus nuclear intensity were calculated. Five random regions in the cytoplasm and nucleus were selected from each cell. At least ten cells were included in each group. Three biologically independent replicates were performed. Data are presented as mean values ± SD. *P* values are calculated by unpaired two-tailed Student’s *t* tests. **c** Western blotting of cytoplasmic and nuclear BARD1 and BRCA1. U2OS cells expressing GFP-BARD1^WT^ and GFP-BARD1 variants were treated by 5 Gy IR and lysed 4 h after IR, followed by the separation of the cytoplasmic and nuclear protein fractions. The factions were respectively loaded for western blotting with anti-GFP antibody or anti-BRCA1 antibody. Type II topoisomerases (TopoII) was used as a nuclear protein loading control; β-actin was used as a cytoplasmic protein loading control. **d** Cytoplasmic percentage of BARD1 and BRCA1 in U2OS cells. The relative intensity of the western blot bands for BARD1 and BRCA1 were quantified, and the percentages of cytoplasmic protein/cytoplasmic+nuclear protein in different BARD1 mutant cells were calculated. Three biologically independent replicates were performed. Data are presented as mean values ± SD. *P* values are calculated by unpaired two-tailed Student’s *t* tests. Source data are provided as a Source Data file.

Although the R378S mutation did not significantly affect BARD1 foci formation (Fig. 2a–c), we noticed that, after DNA damage, almost all GFP-BARD1^WT^ was distributed in the nucleus together with BRCA1 (4 h after IR), whereas a weak GFP-BARD1^R378S^ signal could be detected in the cytoplasm (Fig. 4a, b). To confirm the cytoplasmic BARD1^R378S^, cells were separated into nuclear and cytoplasmic contents followed by western blot. As shown in Fig. 4c, almost all GFP-BARD1^WT^ was present in the nucleus at 4 h after IR treatment, whereas there was a higher cytoplasmic-nucleus ratio of the BARD1^R378S^ variant. On average, the R378S mutation caused the retention of ~10–14% of BARD1^R378S^ in the cytoplasm. As expected, a small amount of endogenous BRCA1 was detected in cytoplasm in the BARD1^R378S^ cells (Fig. 4c, d). This finding is supported by that of a previous study in which nuclear targeting of BARD1 was systematically mapped and analyzed^19^. Notably, R378 is seated within the fourth nuclear localization signal (NLS4, aa 364–380) of BARD1, which was found to be a novel NLS partially mediating BARD1 nuclear import^19^. The results suggest that although the R378S mutation does not give rise to notable defects in DDR or cell fate, it partially affects the nuclear localization of BARD1^R378S^ and BRCA1.

### BARD1^P24S/R378S^ has disrupted DNA repair capacity and causes genome instability

**Fig. 5:**
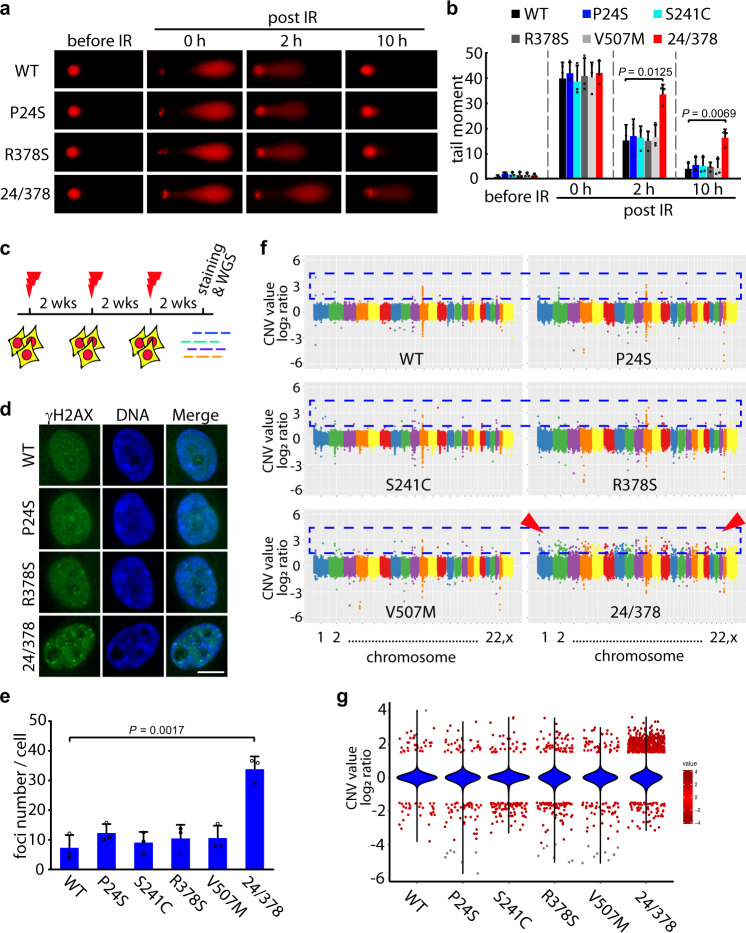
P24S/R378S double mutation causes genome instability. **a** Comet assay of MCF10A cells expressing WT BARD1 or different BARD1 variants. IR-treated cells at different time points were harvested and subjected to neutral comet assays. **b** Comet assay summary of MCF10A cells from **a**. Tail moments of cells represent data from three independent experiments, with at least 30 cells in each group. Data are presented as mean values ± SD. *P* values are calculated by unpaired two-tailed Student’s *t* tests. **c** Experimental design for the IR/recovery experiment. MCF10A cells expressing WT BARD1 or different BARD1 variants were treated with three rounds of IR (5 Gy) followed by a 2-week recovery phase after each treatment. After the third round of recovery, cells were collected for subsequent staining or whole-genome sequencing (WGS). **d** Immunofluorescence staining of γH2AX in MCF10A cells expressing different BARD1 variants. The treated cells from **c** were fixed and stained with an anti-γH2AX antibody and DAPI. Scale bar, 10 μm. **e** Number of γH2AX foci in cells from **d**. The foci number in individual cells expressing BARD1^WT^ or different BARD1 variants was counted. At least 30 cells were included for each group. Three biologically independent replicates were performed. Data are presented as mean values ± SD. *P* values are calculated by unpaired two-tailed Student’s *t* tests. **f**, **g** Whole-genome CNV analysis of MCF10A cells expressing BARD1^WT^ or different BARD1 variants. Genomic DNA of the treated cells from **c** was extracted and subjected to WGS, followed by CNV analysis with CNV-Seq. The CNV value of each chromosome (1–22, and X) in different cell groups is shown as the log2 ratio of **f**. Total CNV value for all chromosomes in the different cell groups are summarized in **g**. Violins denote the status of total DNA, and scatters denote DNA segments with absolution log2 ratio >1.5. Source data are provided as a Source Data file.

To figure out whether the BARD1^P24S/R378S^ variant has a substantial effect on cell fate and especially on genome instability, we examined global DNA damage by comet assay. Non-transformed breast cells MCF10A with endogenous BARD1 knockout and stably expressing BARD1^WT^ or different BARD1 variants were treated by IR and recovered for hours. IR caused obvious genomic lesions to WT and all BARD1-mutated cells (post IR 0 h). After 10 h of recovery, cells expressing WT and single mutated BARD1 variants repaired their damage and the comet tails nearly disappeared. Although in cells bearing BARD1^P24S/R378S^, obvious damage still existed (Fig. 5a, b), suggesting an attenuated ability of DNA repair in these cells.

To validate the effect of the P24S/R378S double mutation on genomic stability, we treated these cells with three rounds of IR and allowed them to recover for 2 weeks after each IR hit, mimicking a long term of tumorigenesis from non-transformed cells (Fig. 5c). After the last round of recovery, cells (WT, P24S, S241C, R378S, V507M, and P24S/R378S BARD1 groups) were harvested followed by DNA damage staining and whole-genome sequencing. Of note, cells bearing BARD1^P24S/R378S^ contained obvious DNA lesions marked by phosphorylated H2AX (γH2AX) (Fig. 5d, e). In parallel, whole-genome sequencing (10×, ~30 GB/sample) was performed followed by CNV analysis. Cells expressing WT BARD1 without IR treatment were used as a control for comparison. The log2 ratio reflecting the CNV level of each cell line (WT, P24S, S241C, R378S, V507M, and P24S/R378S BARD1 groups) after IR/recovery compared with the control was calculated for each chromosome. As shown in Fig. 5f, the three rounds of IR/recovery did not cause obvious CNVs in cells bearing BARD1^WT^, BARD1^P24S^, BARD1^S241C^, BARD1^R378S^, or BARD1^V507M^. However, cells bearing BARD1^P24S/R378S^ contained significantly more CNVs, which involved all chromosomes (Fig. 5f). All CNVs of each cell group are summarized in Fig. 5g. These results indicate that the BARD1 double-mutant P24S/R378S causes genome instability and potentially promotes tumorigenicity of cells.

### BARD1^P24S/R378S^ promotes tumorigenesis in vivo

**Fig. 6:**
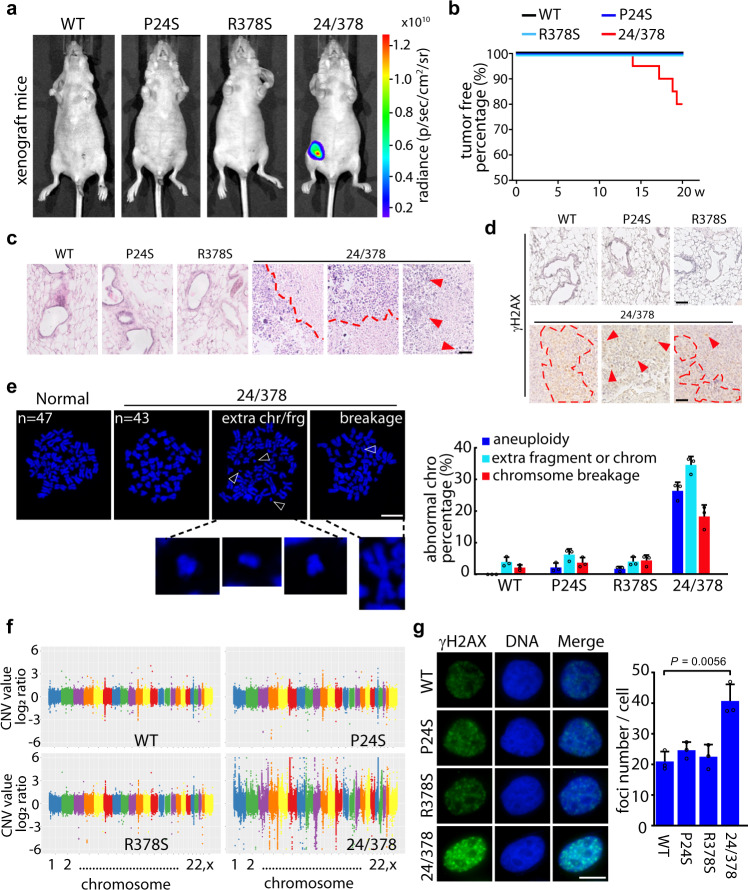
P24S/R378S double mutation promotes tumorigenesis in mice. **a** In vivo imaging of mice bearing xenografts of MCF10A cells harboring different BARD1 variants. Luciferase-MCF10A cells expressing WT or different BARD1 variants combined with *p53* knockout were transplanted into the fourth mammary fat pad of BALB/c nude mice (aged 4–6 weeks). Tumor growth in mice was quantified by bioluminescence imaging. **b** Kaplan–Meier analysis of tumor-free percentage of xenograft mice. Tumor growth of xenograft mice from **a** was observed and analyzed by Kaplan–Meier survival curve. Twenty mice were included in each group. **c** H&E staining of mammary tissue of xenograft mice. MCF10A cells bearing BARD1^WT^, BARD1^P24S^, or BARD1^R378S^ caused no cancerous lesion in mammary gland, whereas the cells bearing BARD1^P24S/R378S^ developed tumors. Obvious tumor regions are denoted by dashed lines or arrowheads. Scale bar, 50 μm. **d** γH2AX staining of mammary tissue from xenograft mice. Massively γH2AX positive signals were found in mammary tissue from mice bearing BARD1^P24S/R378S^ cells. Typical positive stains are denoted by dashed or arrowheads. Scale bar, 50 μm. **e** Representative mitotic spreading of MCF10A cells. MCF10A cells expressing BARD1^P24S/R378S^ were isolated from tumor mass and subjected to mitotic spreading analysis. Representative aneuploidy, extra chromosome or fragment, and chromosome breakage phenotypes from BARD1^P24S/R378S^ cells are shown. The percentages of abnormal chromosome types in BARD1^WT^ and BARD1 variant cells are summarized in the histogram. At least 30 cells were included for each group. Three biologically independent replicates were performed. Data are presented as mean values ± SD. Scale bar, 10 μm. **f** Whole-genome CNV analysis of MCF10A cells with WT *p53* background expressing BARD1^WT^ or different BARD1 variants re-harvested from xenograft mice three weeks after transplantation. Genomic DNA of the re-harvested cells from individual mouse was extracted and subjected to WGS, followed by CNV analysis with CNV-Seq. Three transplanted mice were used for each cell type group. A representative picture of CNV value of chromosomes (1–22, and X) for each group is shown as the log2 ratio. **g** Immunofluorescent staining of γH2AX of MCF10A cells expressing BARD1^WT^ or different BARD1 variants re-harvested from xenograft mice 3 weeks after transplantation. Scale bar, 10 μm. The γH2AX foci number in each cell was counted. At least 50 cells were included for each group. Three biologically independent replicates were performed. Data are presented as mean values ± SD. *P* values are calculated by unpaired two-tailed Student’s *t* tests. Source data are provided as a Source Data file.

To assess the effect of BARD1^P24S/R378S^ on tumorigenesis in vivo, luciferase-MCF10A cells were reconstructed with BARD1^WT^, BARD1^P24S^, BARD1^R378S^, or BARD1^P24S/R378S^, combined with p53 knockout (Supplementary Fig. 3). These cells were pretreated with three rounds of IR/recovery as Fig. 5c and transplanted into mammary fat pad of female BALB/c nude mice followed by monitoring the progress of tumor growth. No detectable tumors were found in the xenograft mice bearing WT or single-mutant BARD1 cells during the 20 weeks of observation. However, 4 out of 20 transplanted mice in BARD1^P24S/R378S^ group developed tumors (Fig. 6a, b). H&E staining revealed that cells expressing WT or single-mutant BARD1 did not survive in vivo and underwent degradation. The local tissue showed normal alveoli and stroma structures. In contrast, the BARD1^P24S/R378S^ cells succeeded in colonizing and developing into a tumor mass within the local mammary gland and were marked by prominent nucleoli with scant cytoplasm and high proliferation (Fig. 6c). When testing the extent of DNA damage, mammary gland tissues transplanted with cells expressing WT or single-mutant BARD1 had no detectable DNA lesions in, whereas tumor masses with cells bearing BARD1^P24S/R378S^ had many DNA breaks marked by γH2AX (Fig. 6d). To further confirm the presence of genome instability, live cells were isolated from the tumor masses for chromosome spreading analysis. As MCF10A cells bearing WT BARD1 or single-mutant BARD1 did not develop tumors and underwent degradation in vivo, we could not obtain them from the xenograft mice. Corresponding cells cultured in parallel in vitro were thus used for chromosome spreading analysis. As seen in Fig. 6e, most of MCF10A cells bearing WT or single-mutant BARD1 displayed a normal karyotype with 47 chromosomes. However, a fair number of MCF10A cells bearing BARD1^P24S/R378S^ showed aneuploidy (26%), extra fragments or chromosome (34.6%), or chromosome breakage (17.9%) (Fig. 6e).

As p53 deficiency was used as a background for the BARD1 mutant cells to speed tumor growth in the experiments above, we were curious whether the BARD1 double mutations per se could induce genome instability in vivo. MCF10A cells (with WT p53) were reconstructed with BARD1^WT^, BARD1^P24S^, BARD1^R378S^, or BARD1^P24S/R378S^ followed by IR treatment, as illustrated in Fig. 5c. The four types of cells were then transplanted into mice using the same procedure described in Fig. 6a. Three weeks after transplantation, these cells were re-harvested from the mice for accessing genome instability. The re-harvest time point as 3 weeks depended on the longest survival lifetime for all the four types of cells because the BARD1^WT^ cells degraded or were absorbed by surrounding tissue in vivo in the fourth week after xenografting. Next, we performed whole-genome sequencing (10×, ~30 GB/sample) of these cells and profiled their genome instability. As shown in Fig. 6f (also Supplementary Fig. 3b, c) the number of CNVs in BARD1^P24S/R378S^ cells was significantly higher than that in BARD1^WT^, BARD1^P24S^, and BARD1^R378S^ cells, and involved all chromosomes. Moreover, DNA damage in the re-harvested cells was detected by immunofluorescent staining of γH2AX. Consistent with the higher number of CNVs, the cells bearing BARD1^P24S/R378S^ contained more severe DNA lesions than the other groups of cells (Fig. 6g). These data suggest that the P24S/R378S double mutations of BARD1 results in high genomic instability that could lead to tumorigenesis in vivo.

## Discussion

As a crucial tumor suppressor, BRCA1 functions in DNA damage repair and maintains genome integrity^5,6^. Dysfunction of BRCA1 leads to a high risk of breast and ovarian cancer for women^8–10^. However, it is very challenging to identify the exact pathogenic mutations related to BRCA1 dysfunction and tumorigenesis owing to interference by large numbers of detected variations, and the gap between clinical resources and molecular functions. In this study, we identified four mutations in BARD1 but none in BRCA1 in a screen of an HBOC family. None of the individual mutations affected DDR or cell fate. Intriguingly, two mutations, i.e., P24S and R378S, simultaneously existing in cis in both of the surviving cancer patients in the family, jointly impaired DDR. After recovery from treatment with a DNA damage agent, cells bearing the double-mutant BARD1^P24S/R378S^ but not the single-mutant BARD1 variants showed obvious genomic instability, and developed into tumors in a xenograft mouse model. These findings highlight the synergetic effect of cis mutations in individual genes on tumorigenesis, as well as an attractive target of BARD1 but not BRCA1 per se for the treatment of BRCA1-associated tumors.

Both mice of mammary epithelial-specific ablation of Bard1 and Brca1 develop breast tumor resembles human triple-negative breast cancer with the same kinetics^50^. This finding suggests an equivalent relationship between these two partners and the significant roles of BARD1 in tumor suppression. Unlike BRCA1, whose biological functions have been well explored^6,20,51^, the regulatory mechanism of BARD1 is far from clear. The P24 and R378 amino acids lie in the N-terminus and middle of BARD1, respectively. It is reported that amino acids 26–110 of BARD1 is the core region for binding BRCA1^16^, of which P24 locates outside. This may explain why the P24S mutation only leads to a biochemical change in vitro rather than a detectable cellular defect or tumorigenesis in vivo. However, combined with other factors, this type of “mild” mutation may give rise to a severe phenotype. The R378S mutation also did not cause a significant cellular phenotype, but attenuated nuclear retention of the BRCA1/BARD1 complex upon DNA damage. This weak phenotype can be explained by the fact that six NLSs have been identified in full-length BARD1^19,52,53^, and the R378-containing fourth NLS contributes partially to BARD1 nuclear location. The multiple NLSs in BARD1 further imply the complicated regulation of this protein.

Over the past few years, comprehensive sequencing efforts have uncovered a large number of mutations across clinical samples^54–56^. Only a small fraction of these mutations have been definitely proven as carcinogenic mutations, whereas the others are classified as unidentified variations that require in-depth investigation. Herein, we conclude that certain non-pathogenic single variant (e.g., P24S) of a given gene, when combined with another variant (R378S) in cis, can yield a pathogenic allele. This synergetic effect would be more likely to occur in any gene that carries two or more functional domains or regions. For these genes, synergetic effects may be involved in a significant fraction of all tumor cases and thus have important clinical implications. Thus, a large number of variants, whose effects were previously uncertain, should be reevaluated. Taking C557S of BARD1 for example, several population screens have shown that this variation is associated with breast and ovarian cancers^57–59^. In contrast, other studies did not find a correlation of C557S with cancer^60–62^. These inconsistent results do not seem to be caused by the analysis methods, but may indeed be influenced by the existence of another cis variant that could generate a synergetic effect with C557S. Similarly, the BRCA1 protein contains at least two well-known domains, namely the N-terminal RING and C-terminal BRCTs, that contribute distinct functions to BRCA1^6^. And thus, mutations localized in the two regions in cis in *BRCA1*, and also in many other genes, should be studied and reported for the early diagnosis of tumors.

## Methods

### Ethics

All human materials used in this study were approved by Peking University Third Hospital Medical Science Research Ethic Committee (IRB00006761-M2019343). Signed informed consents were obtained from the family members who participated in the study. Mice care and handling were conducted in accordance with policies promulgated by the Ethics Committee of Peking University Health Science Center.

### Chemicals and antibodies

All chemicals were purchased from Sigma except for those specifically mentioned. Anti-BARD1 (ab226854), ER (ab16660), and β-actin (ab8226) antibodies were purchased from Abcam. Anti-PR (8757), HER2 (2165), TOPOIIα (12286 s), GFP (2956), γH2AX (2577 S), and phospho-histone H3 (Ser10) (9701 s) antibodies were purchased from Cell Signaling Technology. Anti-BRCA1 (sc-6954) and P53 (NB200-103) antibodies were purchased from Santa Cruz Biotechnology and Novus, respectively. Alexa FluorTM 633 goat anti-mouse IgG (A-21126), Alexa FluorTM 555 goat anti-Rabbit IgG (A-21429), HRP goat anti-mouse IgG (H + L) secondary antibody (32430), and HRP goat anti-rabbit IgG(H + L) secondary antibody (31466) were purchased from Thermo Fisher Scientific. Further information of the dilution of each antibody was listed in the reporting summary section.

### DNA preparation and WGS

Mononuclear cells were isolated by Ficoll-Paque from peripheral blood samples obtained from the participants. The genomic DNA of the cells was extracted and purified by QIAamp^®^ DNA Blood Mini kit (QIAGEN) according to standard protocol. DNA quality was assessed by gel electrophoresis and Qubit® 2.0 Flurometer (Life Technologies, CA, USA). In all, 1 μg of genomic DNA per sample was used for library construction with TruSeq DNA Sample Preparation Guide (Illumina, 15026486 Rev.C). The qualitied DNA libraries were sequenced using Illumina Whole Genome Sequencing Service with Illumina HiSeq X at the Core Genomic Facility of Beijing Annoroad Genomics. All data were aligned to hg19 with BWA (Burrows-Wheeler Alignment tool)^63^, arranged with SAMtools (Sequence Alignment/Map tool)^64^, marked with Picard (http://broadinstitute.github.io/picard/), locally aligned with GATK (Genome Analysis Toolkit)^65^. Variants were annotated using ANNOVAR tool (Annotate Variation)^66^.

### Plasmid construct and CRISPR knockout

*BARD1* (full length, 2331 bp, 777aa) was cloned into pEGFP-C1 vector. The mutations (P24S, S241C, R378S, V507M, and P24S/R378S) were generated using the QuikChange site-directed mutagenesis kit (TransGen Biotech). For GST tag fusion protein expression, BARD1-N (1–142 aa) and BRCA1-N (1–147 aa) were cloned into pGEX-4T-1 vector. For knockout of endogenous *BARD1* and *p53* by CRISPR/Cas9 system, two guide RNAs for each gene were designed at https://zlab.bio/guide-design-resources. The *BARD1* gRNAs are: gRNA-1 (exon1): GTCGAGCGCGGCGCGACTGTGGG; BARD1 gRNA-2 (exon4): ATCTGACTTTCTTACTTCGAGGG. The *p53* gRNAs are:

P53 gRNA-1 (exon1): TCGACGCTAGGATCTGACTGCGG; P53 gRNA-2 (exon3): CCATTGTTCAATATCGTCCGGGG. All the primers used in this work were listed in Supplementary Data 5. Lentiviral expressing CRISPR-Cas9 vector generated by Feng Zhang lab, plentiCRISPRv2, was used for knockout. This one-vector system expresses the gRNA, Cas9 protein, and puromycin resistance gene from one virus, constructed by https://media.addgene.org/cms/files/Zhang_lab_LentiCRISPR_library_protocol.pdf.

### LOD score calculation

The *LOD* function in R package *paramlink* (version 1.1–2, https://CRAN.R-project.org/package=paramlink) was used to calculate LOD score. In specific, the locus for R378 (wild type) was denoted as “A”, and the corresponding mutated locus for S378 as “a”; while wild type P24 as “B”, and the mutated S24 as “b”. The four haplotypes of “AB”, “Ab”, “aB”, and “ab” were further assigned the codes of “1”, “2”, “3”, and “4”, respectively, for calculating LOD score in R package *paramlink*. Since the two polymorphisms are linked on the same allele, based on the sequencing data we could confirm the genotypes of the five female members in proband generation, i.e., the five females (#11, #12, #15, #17, #19) are: AB/ab (1/4), aB/ab (3/4), AB/AB (1/1), AB/AB (1/1), and aB/ab (3/4), respectively. According to Mendel’s law, we deduced genotypes of the members of previous generations (#4, #5, #6, #7, #8, #9, #10; #2) that are required for LOD score calculation. The uncertain haplotype of the family member was replaced with asterisk (*). Thus, the genotypes and the codes of #4, #5, #6, #7, #8, #9, #10, and #2 are: */ab (*/4), AB/* (1/*), AB/* (1/*), AB/ab (1/4), AB/* (1/*), */ab (*/4), */aB (*/3), and */ab (*/4). For * haplotype, we randomly assigned any possible haplotype for running LOD calculation program in R package *paramlink*. Besides, considering the difference pathogenesis between genetic disease and cancer, the AFF value (Affection status, 1=unaffected, 2=affected, 0=unknown as in “Linkdat” format data, for details please see https://www.rdocumentation.org/packages/paramlink/versions/0.7-0/topics/linkdat) of the “health for now” Person-12 was identified as “0”. Because of the male gender of Person-4 that has extremely low or no possibility for the occurrence of breast or ovarian cancers, the AFF value was also identified as “0”. The “Linkdat” format data of the pedigree is provided in Supplementary Data 6. The LOD score was calculated with the following commands, in which the *dataped.txt* is the same as Supplementary Data 6:

$$dataped = read.table\;(\prime dataped.txt,\;header = T)$$

$$x = linkdat\;(dataped,\;model = 1)$$

$$res = lod\left( x \right).$$

### Histological staining

Histological staining was performed at the Immunohistochemistry Core of Peking University Third Hospital. Tissues were fixed in 10% neutral-buffered formalin solution for 12–16 h and gradually transferred to 70% ethanol. After embedded in paraffin, the tissues were cut in 5 μm sections on polylysine-coated slides and stained with H&E, or indicated antibodies. The dilutions of anti-ER, PR, HER2, and P53 antibodies are 1:250, 1:500, 1:400, and 1:250, respectively. Images were taken and analyzed by Olympus BX51 microscope and DP73 CCD photographic system.

### Cell culture, live imaging, and IR treatment

U2OS cells were cultured in Dulbecco’s Modified Eagle Medium (DMEM) with 10% fetal bovine serum (FBS). MCF10A cells were cultured in DMEM/nutrient mixture F-12 with 5% FBS, 1× ITS, 0.02 μg/ml EGF, 0.5 μg/ml hydrocortisone, and 0.1 μg/ml cholera toxin. For live imaging, cells were grown on imaging culture dish (NEST, 801001) and observed in UltraVIEW VoX (PerkinElmer) live-cell workstation at 37 °C with 5% CO_2_. Images were analyzed by Volocity (Universal 3D Image). For IR treatment, cells were irradiated with a 137Cs source at a dose of 5 Gy (or at indicated doses) and used for subsequent experiments.

### G2/M checkpoints assay

Cells expressing WT or mutant BARD1 variants were treated with or without 2 Gy of IR. After 1 h of recovery, cells were fixed with 70% (v/v) ethanol, stained with rabbit antibody to phospho-histone H3 (pSer10), and then incubated with fluorescence-conjugated goat secondary antibody against rabbit. The stained cells were treated with RNase A and then dyed with propidium iodide. Samples were analyzed by flow cytometry (BD FACSCelesta) with FlowJo V10 software. Flow cytometry gating strategy was provided in Supplementary Fig. 4.

### Microscale thermophoresis

MST was performed according to our previous work as described^47^. In brief, purified protein of GST-BARD1-N or GST-BARD1^P24S^-N was labeled with a RED-NHS protein labeling kit (NanoTemper, Germany) according to standard protocol. For semi-in vivo assay, fluorescent GFP-BARD1^WT^ or GFP-BARD1^P24S^ in cell lysate was normalized by raw fluorescence [counts] and then directly used for subsequent steps. The labeled (or fluorescent) protein was then incubated at a constant concentration (10–50 nM) with twofold serial dilutions of GST-BRCA1-N in MST optimized buffer (50 mM Tris-HCl pH 7.4, 150 mM NaCl, 10 mM MgCl2, 0.05% Tween 20). Equal volumes of binding reactions were mixed by pipetting and incubated for 15 min at room temperature. Mixtures were enclosed in standard-treated or premium coated glass capillaries and loaded into the instrument (Monolith NT.115, NanoTemper, Germany). Measurement protocol times were as follows: fluorescence before 5 sec, MST on 30 sec, fluorescence after 5 sec, delay 25 sec. For all the measurements, 200–1000 counts were obtained for the fluorescence intensity. The measurement was performed at 20% and 40% MST power. Fnorm = F1/F0 (Fnorm: normalized fluorescence; F1: fluorescence after thermodiffusion; F0: initial fluorescence or fluorescence after T-jump). *Kd* values were determined by the NanoTemper analysis tool.

### MD simulation

The solution NMR structure of the BRCA1/BARD1 RING-domain heterodimer^67^ (PDB ID: 1JM7) was used for the MD simulations. As the residues prior to M26 in BARD1 were missing in the resolved structure, six residues from full-length template (residues 20–25 aa of BARD1) were added at the N-terminal of BARD1 with Modeller^68^ to include the residue of interest, P24. The P24S mutant model was constructed in a similar way by replacing the wild-type P24 with S24. The BRCA1/BARD1 structure was solvated in a solution of 150 mM KCl to neutralize the system, resulting in a simulation box of ~9.3 × 9.1 × 8.5 nm^3^. First, 5000-step energy minimization was conducted to remove the bad contact in the systems. Then, the systems were equilibrated in a 0.5-ns simulation under a canonical (NVT) ensemble and a 1-ns simulation under an isothermal–isobaric (NPT) ensemble, with the heavy atoms of the proteins restrained to their initial positions. After the above relaxation and equilibration, the restraints were released and 500-ns production simulations were performed for both the wild type and mutant systems. All the simulations were performed with the AMBER99SB force field^69^ and SPC/E water model^70^ with a time step of 2 fs. The Berendsen thermostat and pressure coupling^71^ were used for the NPT equilibration, and the v-rescale thermostat^72^ and Parrinello–Rahman pressure coupling^73^ were used for the production simulations. The electrostatic interactions were calculated with the Particle Mesh Ewald method^74^. The Van der Waals interactions were calculated within a cutoff of 1 nm. Gromacs 2018.6^75^ was used for running the MD simulations and trajectory analysis.

### Immunofluorescence

Cells were fixed in 4% paraformaldehyde in PBS (pH 7.4) for 30 min followed by permeabilization in 0.5% Triton-X-100 for 25 min at room temperature. Then, samples were blocked with 1% bovine serum albumin-supplemented PBS for 1 h and incubated with the indicated primary antibodies (1:200–1:500) at 4 °C overnight. After washing three times in PBS containing 0.1% Tween 20 and 0.01% Triton-X 100, cells were incubated with an appropriate fluorescent secondary antibody for 1 h at room temperature. After washing three times, cells were stained with Hoechst 33342 (10 µg/ml) for 15 min. Finally, samples were mounted on glass slides and observed under a confocal laser scanning microscope at ×63/1.40 (Carl Zeiss 710).

### Ubiquitin ligase activity assay

Ubiquitin ligase activity assay was performed according to previous reports^23,41^. In brief, N-terminal fragment of BRCA1 (1–300 amino acids) that exhibit ubiquitin ligase activity, and full length of wild-type and mutant BARD1 were fused expressed with GST tag followed by protein purification. Ubiquitin-conjugation reaction was set up by adding 1 μM E1, 4 μM E2 (Ube2d3), 8 μM BRCA1-N, 8 μM BARD1 (WT or mutant), 20 μM Ub, 0.12 μM mono-nucleosomes, 5 mM ATP, and 5 mM MgCl_2_. The mixture solution was incubated at 37 °C with shaking for 1 h. Then the reaction solution was subjected to SDS electrophoresis followed by western blot detection against ubiquitin antibody.

### Western blot

Total protein was extracted from cell lysate by radioimmunoprecipitation buffer. For nuclear and cytoplasmic proteins, the fractions were separated with EMD Millipore Nuclear Extraction Kit (Catalog no. 2900) according to standard protocol. Protein samples were separated by SDS-polyacrylamide gel electrophoresis and then electrically transferred to polyvinylidene fluoride membranes. Following transfer, the membranes were blocked in tris-buffered saline with Tween 20 (TBST) containing 5% skimmed milk for 2 h, followed by the incubation with indicated primary antibodies overnight at 4 °C. After washing in TBST three times, the membranes were incubated at 37 °C for 1 h with 1:1000 dilution of horseradish peroxidase-conjugated secondary antibody. Finally, protein bands were visualized by enhanced chemiluminescence detection system (Amersham Biosciences).

### Comet assay

Single-cell gel electrophoretic comet assay was performed under neutral conditions to detect DSBs according to the previous study^76^. In brief, cells with or without indicated treatment were recovered in normal culture medium. Then cells were collected and rinsed twice with ice-cold PBS. In all, 2 × 10^4^/ml cells were combined with 1 % LMAgarose at 40 °C at the ratio of 1:3 (v/v) and immediately pipetted onto slides. For cellular lysis, the slides were immersed in the neutral lysis solution (2 % sarkosyl, 0.5 M Na_2_EDTA, 0.5 mg/ml proteinase K in pH 8.0) overnight at 37 °C in dark, followed by washing in the rinse buffer (90 mM Tris-HCl pH 8.5, 90 mM boric acid, 2 mM Na_2_EDTA) for 30 min with two repeats. Then, the slides were subjected to electrophoresis at 20 V (0.6 V/cm) for 25 min and stained in 2.5 µg/ml propidium iodide for 20 min. All images were taken with a fluorescence microscope (Olympus IX73) and analyzed by Comet Assay IV software.

### CNV analysis

MCF10A cells were reconstructed with WT BARD1 or different BARD1 variants (P24S, S241C, R378S, V507M, or P24S/R378S). After IR treatments and recoveries, genome DNA of the cells were extracted and prepared as above in “DNA Preparation and Whole Genomic Sequence” followed by WGS (10×, ~30 GB/sample). After aligning clean paired-end sequencing reads to the hg38 human reference genome (UCSC) using burrows-wheeler aligner (BWA), the duplicated reads were removed using Picard. CNV analysis was performed with CNV-seq as described previously^77^. In brief, all mapped reads were converted from *.bam files to best-hit location files by SAM tools. The best-hit location files were then subjected to a cnv-seq.pl script with the default parameters to generate the putative CNVs (–test, MCF10A cells bearing WT or different BARD1 variants with IR treatment and recovery;–ref, MCF10A cells bearing WT BARD1 without IR treatment). Data were plotted in R using the ggplot2 package.

### In vivo imaging

Luciferase-MCF10A cells in which endogenous BARD1 and p53 were knockout were reconstructed with WT or different BARD1 variants. In all, 2 × 10^6^ cells in a volume of 100 μl (1:1 mixture of PBS and Matrigel) were injected into the fourth mammary fat pad of female BALB/c nude mice aged 4–6 weeks. Tumor growth was monitored weekly by bioluminescence imaging with the IVIS Spectrum (PerkinElmer). During the monitor, mice were gas anesthetized with isofluorane (2% isoflurane in 100% oxygen, 1 L/min). In total, 200 μl d-luciferin at 10 mg/ml was administered by intraperitoneal injection 10 min before live imaging. Signals of images were quantified and analyzed by Living Image 3.10 software.

### Chromosome spreading

Primary cells from tumor masses in xenograft mice were isolated. The cells were synchronized to mitotic stage with nocodazole (50 ng/ml) for 12 h and released into fresh medium. The mitotic cells were harvested by mitotic shake-off and centrifuge (100 g, 5 min). After hypotonic incubation in 75 mM KCl for 30 min at 37 °C, cells were fixed in Carnoy solution (75% methanol, 25% glacial acetic acid). The fixed cells were dropped onto slides, dried in air, and stained with Hoechst 33342 in the dark. After briefly washing and dry, the slides were mounted with antifade mounting media and observed with a confocal microscope (Carl Zeiss 710).

### Statistical analyses

All experiments were performed in triplicates unless indicated otherwise. Means and standard deviations were plotted. Student’s *t* test was used for statistical analyses (*P* values were calculated by unpaired two-tailed Student’s *t* tests.). Statistical details are included in figures and figure legends.

### Reporting summary

Further information on research design is available in the Nature Research Reporting Summary linked to this article.

## Supplementary information

Supplementary Information

Description of Additional Supplementary Files

Supplementary Data 1

Supplementary Data 2

Supplementary Data 3

Supplementary Data 4

Supplementary Data 5

Supplementary Data 6

Reporting Summary

## Data Availability

The WGS data reported in this study have been deposited in the genome sequence archive of Beijing Institute of Genomics, Chinese Academy of Sciences, Beijing, China (gsa.big.ac.cn, accession no. CRA002326). The solution NMR structure of the BRCA1/BARD1 RING-domain heterodimer is available from http://www1.rcsb.org/structure/1JM7. The remaining data are available within the Article, Supplementary information or available from the authors upon request. Source data are provided with this paper.

## Source data

Source Data

## Code availability

All custom code and scripts are available from the authors upon request.
