# Supplementary material for: A synergetic effect of BARD1 mutations on tumorigenesis
Source: Nat Commun. 2021 Feb 23;12:1243. doi: 10.1038/s41467-021-21519-3 (PMC7902612; doi:10.1038/s41467-021-21519-3)
Supplement: Supplementary file 1 — Supplementary Information [file 41467_2021_21519_MOESM1_ESM.pdf]

Supplementary Information for

A synergetic effect of BARD1 mutations on tumorigenesis

Wenjing Li[1,2,3,a], Xiaoyang Gu[1,2,3,a], Chunhong Liu[4], Yanyan Shi[5], Pan Wang[1,2], Na Zhang[1,2], Rui Wu[6], Liang Leng[7], Bingteng Xie[1,2], Chen Song[4,8], Mo Li[1,2,3,*]

*Correspondence: limo@hsc.pku.edu.cn (M.L.)

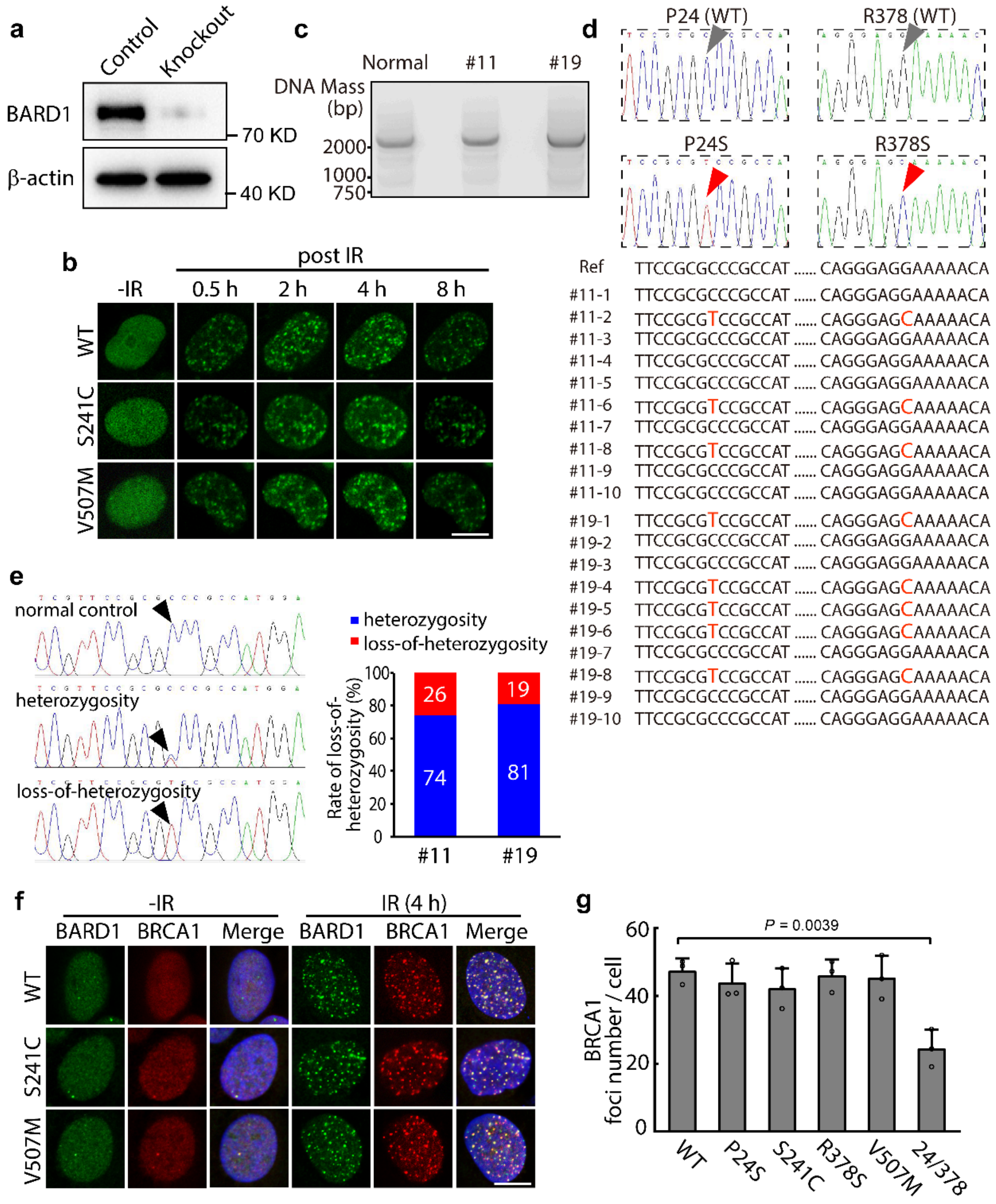

a
Control
Knockout
BARD1
β-actin
70 KD
40 KD
c
Normal
#11
#19
DNA Mass (bp)
2000
1000
750
d
P24 (WT)
R378 (WT)
P24S
R378S
Ref TTCCGCGCCCGCCAT ...... CAGGGAGGAAAAACA
#11-1 TTCCGCGCCCGCCAT ...... CAGGGAGGAAAAACA
#11-2 TTCCGCGTCCGCCAT ...... CAGGGAGCAAAAACA
#11-3 TTCCGCGCCCGCCAT ...... CAGGGAGGAAAAACA
#11-4 TTCCGCGCCCGCCAT ...... CAGGGAGGAAAAACA
#11-5 TTCCGCGCCCGCCAT ...... CAGGGAGGAAAAACA
#11-6 TTCCGCGTCCGCCAT ...... CAGGGAGCAAAAACA
#11-7 TTCCGCGCCCGCCAT ...... CAGGGAGGAAAAACA
#11-8 TTCCGCGTCCGCCAT ...... CAGGGAGCAAAAACA
#11-9 TTCCGCGCCCGCCAT ...... CAGGGAGGAAAAACA
#11-10 TTCCGCGCCCGCCAT ...... CAGGGAGGAAAAACA
#19-1 TTCCGCGTCCGCCAT ...... CAGGGAGCAAAAACA
#19-2 TTCCGCGCCCGCCAT ...... CAGGGAGGAAAAACA
#19-3 TTCCGCGCCCGCCAT ...... CAGGGAGGAAAAACA
#19-4 TTCCGCGTCCGCCAT ...... CAGGGAGCAAAAACA
#19-5 TTCCGCGTCCGCCAT ...... CAGGGAGCAAAAACA
#19-6 TTCCGCGTCCGCCAT ...... CAGGGAGCAAAAACA
#19-7 TTCCGCGCCCGCCAT ...... CAGGGAGGAAAAACA
#19-8 TTCCGCGTCCGCCAT ...... CAGGGAGCAAAAACA
#19-9 TTCCGCGCCCGCCAT ...... CAGGGAGGAAAAACA
#19-10 TTCCGCGCCCGCCAT ...... CAGGGAGGAAAAACA
b
post IR
-IR
0.5 h
2 h
4 h
8 h
WT
S241C
V507M
e
normal control
heterozygosity
loss-of-heterozygosity
heterozygosity
loss-of-heterozygosity
Rate of loss-of-heterozygosity (%)
100
80
60
40
20
0
26
74
19
81
#11
#19
f
-IR
IR (4 h)
BARD1
BRCA1
Merge
BARD1
BRCA1
Merge
WT
S241C
V507M
g
P = 0.0039
BRCA1 foci number / cell
60
40
20
0
WT
P24S
S241C
R378S
V507M
24/378

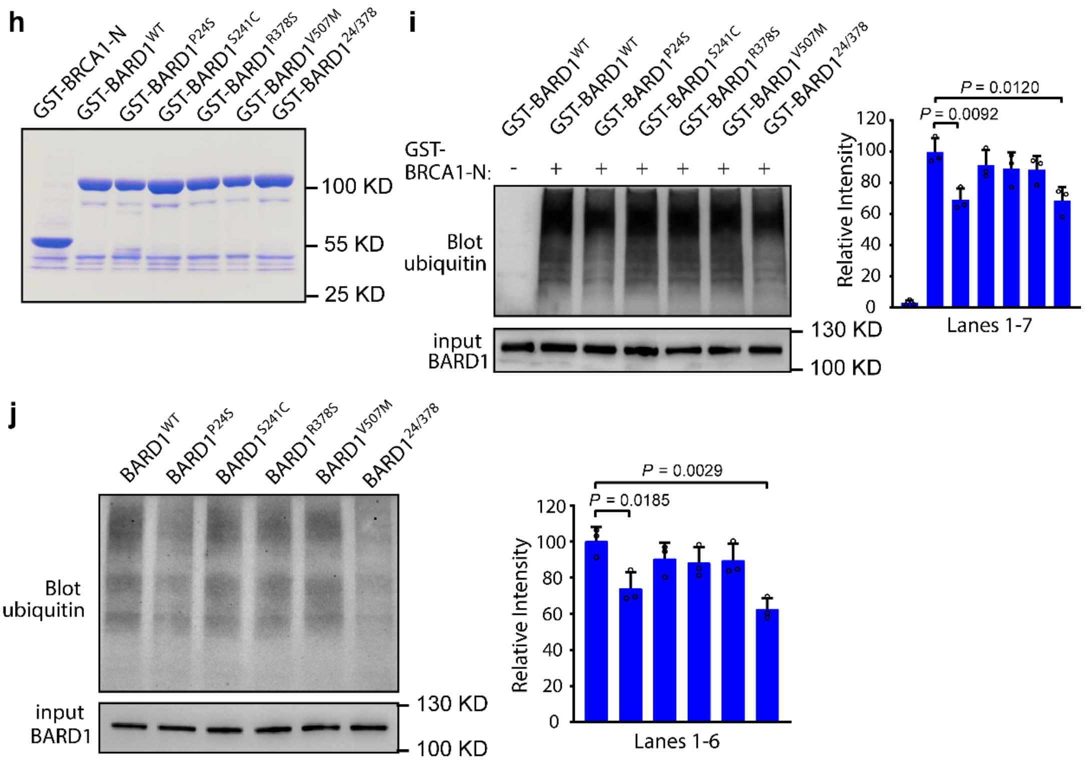

k

	-IR	+IR
WT	1.71±0.09(%)	0.35±0.06(%)
P24S	1.73±0.08(%)	0.34±0.09(%)
S241C	1.61±0.04(%)	0.40±0.05(%)
R378S	1.67±0.12(%)	0.33±0.09(%)
V507M	1.65±0.07(%)	0.29±0.04(%)
P24S/R378S	1.66±0.11(%)	0.89±0.05(%)

Supplementary Fig. 1 Response of different BARD1 variants under DNA damage.

a *BARD1* in U2OS cells was knockout by CRISPR/Cas9. These cells were used for reconstruction by GFP-BARD1^{WT} and the variants. Three biologically independent replicates were performed. **b** Foci formation of BARD1 variants in living cells upon DNA damage. Three biologically independent replicates were performed. Scale bar, 10 µm. **c** PCR amplification of the full length of *BARD1* coding DNA from blood sample of Patient-11 and -19. The blood sample from normal person was used as control. Three biologically independent replicates were performed. **d** *BARD1* coding DNA was sequenced from each monoclonal colony to detect the existence of the mutations of

P24S and R378S. Arrowheads show sequencing data for the two mutations. The mutated bases of each monoclone was labeled in red. **e** Loss-of-heterozygosity in tumor samples was assessed by single-cell sequencing of *BARD1*. 100 single tumor cells were harvested from the tumor mass of each patient followed by single cell whole genome amplification for *BARD1* sequencing. 26 out of 100 cells in the tumor of Patient-11 show homozygous sequence for P24S (loss-of-heterozygosity). While 19 out of 100 cells in the tumor of Patient-19 show loss-of-heterozygosity. Three biologically independent replicates were performed. **f** Immunofluorescence of endogenous BRCA1 in WT or mutant BARD1 cells upon DNA damage. Scale bar, 10 μm. **g** BRCA1 foci number in U2OS cells expressing different BARD1 variants. BRCA1 foci number in each cell expressing GFP-BARD1^{WT}, or GFP-BARD1 variants were counted at the indicated time points after IR treatment. At least 30 cells were included for each group. Three biologically independent replicates were performed. Data are presented as mean values ± SD. *P* values are calculated by unpaired two-tailed Student's *t* tests. **h** N-terminal fragment of BRCA1 (1-300 amino acids) and full length of wild type and mutant BARD1 with GST tag were respectively expressed and purified. The peptide bands were detected by coomassie staining. **i** In vitro reaction of ubiquitin- conjugation was visualized by a formation of ubiquitin chains in western blot against ubiquitin antibody. Levels of ubiquitin-conjugation in different groups were summarized in the histogram. GST-BARD1 in reaction solution was used as input loading control. Three biologically independent replicates were performed. Data are presented as mean values ± SD. *P* values are calculated by unpaired two-tailed Student's *t* tests. **j** U2OS cells

(knockout of endogenous BARD1) stably expressing WT and different mutant BARD1 were treated with 5 Gy IR and lysed. The cell lysates were immunoprecipitated by BRCA1 antibody for SDS electrophoresis followed by western blot against ubiquitin antibody. Levels of ubiquitin-conjugation in different cell groups were summarized in the histogram. GFP-BARD1 in cell lysate was used as input loading control. Three biologically independent replicates were performed. Data are presented as mean values ± SD. *P* values are calculated by unpaired two-tailed Student's *t* tests. **k** G2/M checkpoint activation of U2OS cells expressing different BARD1 variants. Cells were treated with or without IR followed by phospho-histone 3 staining. Cells were examined by flow cytometry and the phospho-histone 3 positive population (*i.e.* mitotic cells) in different cell groups were summarized. Mean percentage and standard deviation are represented.

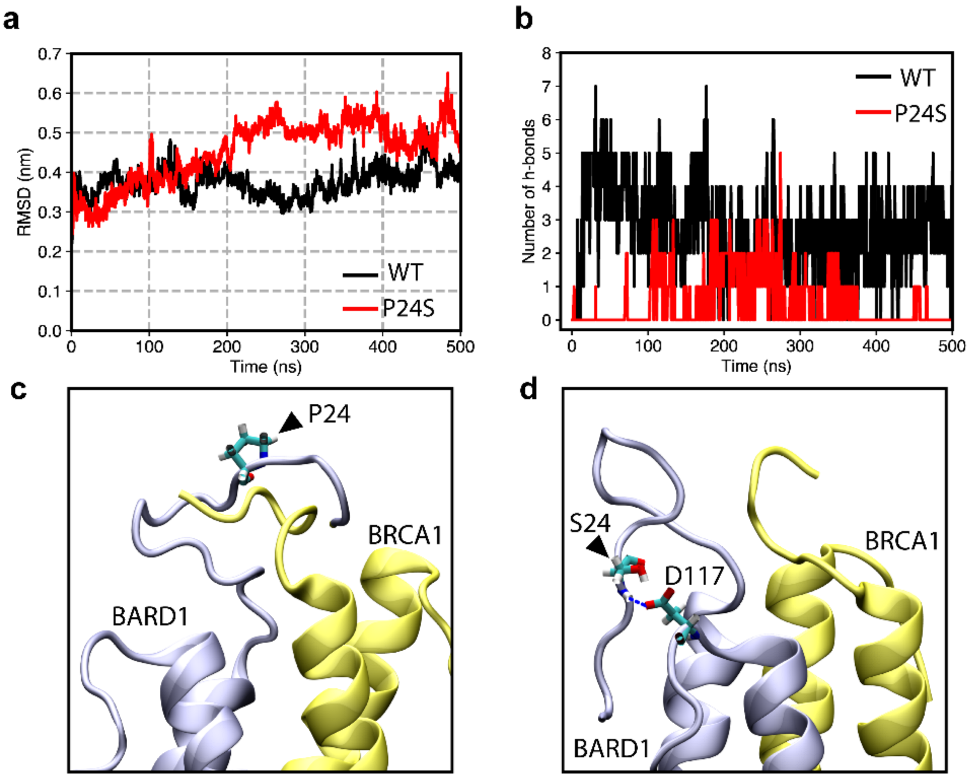

Supplementary Fig. 2 Molecular dynamics simulations of BARD1 and BRCA1. a The RMSD of the alpha carbon atoms in $BARD1^{WT}$/BRCA1 or $BARD1^{P24S}$/BRCA1 RING-domain heterodimer in the MD simulations. The N- and C-terminal coils were not considered for the RMSD calculation due to their high flexibility. **b** The number of hydrogen bonds between the residues 20-28 of BARD1 and the whole BRAC1. The coil region around P24 of $BARD1^{WT}$ can form more hydrogen bonds with BRAC1 than that of $BARD1^{P24S}$. **c, d** Typical conformations around P24 (WT) or S24 (mutant) of BARD1 in the MD simulations. The coil around P24 of $BARD1^{WT}$ tends to interact with BRAC1, while S24 in $BARD1^{P24S}$ tends to form a hydrogen bond with D117 of $BARD1^{P24S}$, prohibiting the interaction between the N-terminal coil of BRCA1 and $BARD1^{P24S}$. BARD1 is shown in ice-blue and BRCA1 is shown in yellow. The key residues are shown as sticks (red: oxygen, cyan: carbon, blue: nitrogen). The hydrogen bond is shown as a blue dashed line. '**c**' and '**d**' were generated with VMD.

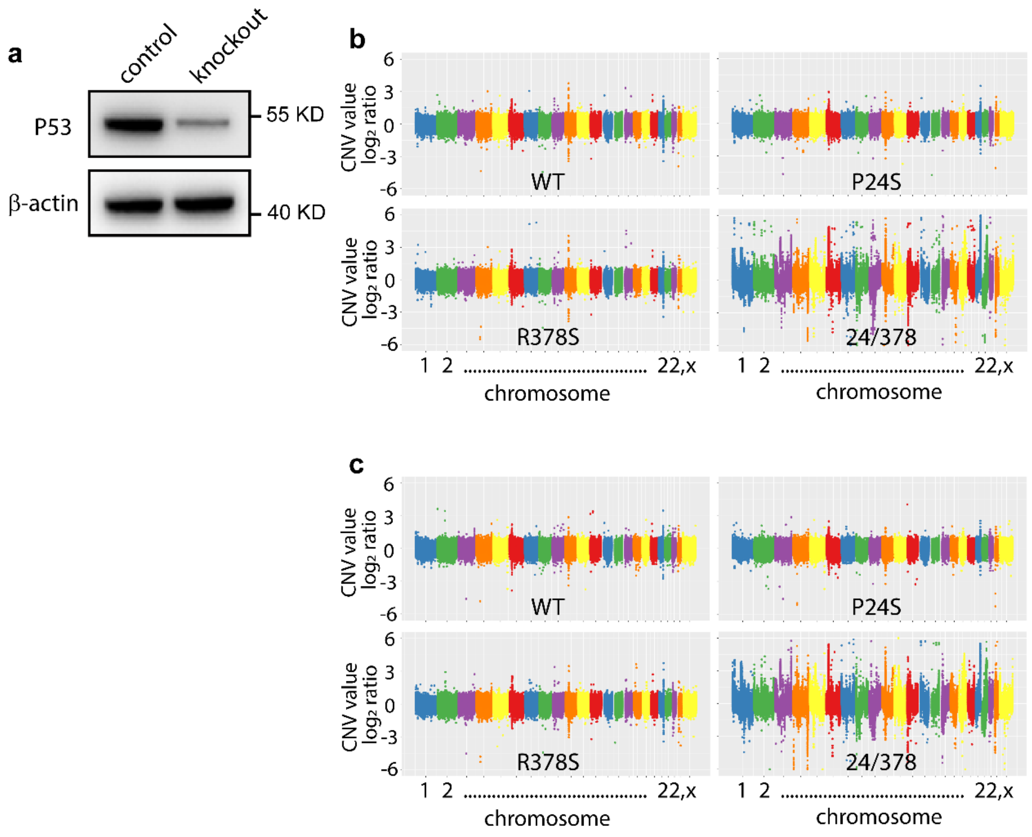

Supplementary Fig. 3 P24S/R378S double mutation causes genome instability in vivo. a Luciferase-MCF10A cells were *p53* knockout by CRISPR/Cas9. These cells were used for reconstruction by WT BARD1 and the BARD1 variants in xenograft mice experiments. The expression of cellular P53 was tested by western blotting with P53 antibody. β-actin was used as loading control. Three biologically independent replicates were performed. **b, c** The other two rounds of replicates of whole genome CNV analysis for Fig. **6f**. MCF10A cells with WT *p53* background expressing $BARD1^{WT}$ or different BARD1 variants were re-harvested from xenograft mice three weeks after transplantation. Genomic DNA of the re-harvested cells from individual mouse was extracted and subjected to WGS, followed by CNV analysis with CNV-Seq. Three transplanted mice were used for each cell type group. CNV value of chromosomes (1- 22, and X) for each group is shown as the log2 ratio.

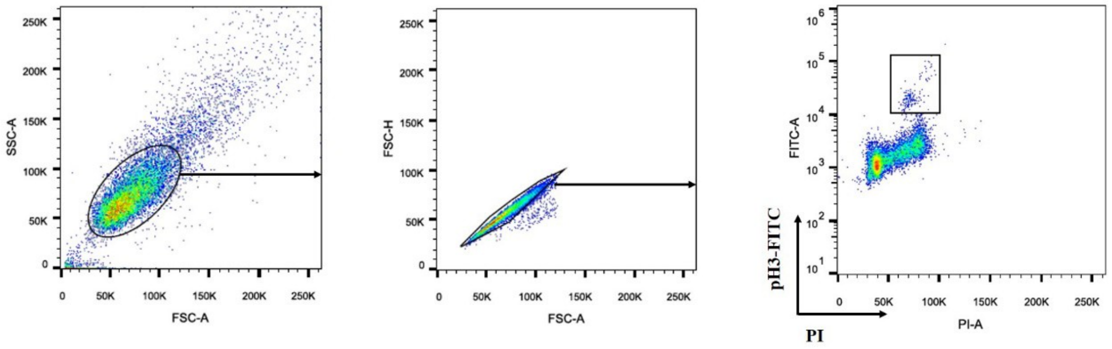

Supplementary Fig. 4 Flow cytometry gating strategy of wild type and BARD1 mutant cells treated with/without IR. Cells were stained with rabbit antibody to phospho-histone H3 (pH3), and then incubated with 488 fluorescence-conjugated goat secondary antibody against rabbit. The stained cells were then dyed with propidium iodide (PI). For gating: at first, forward versus side scatter (FSC vs SSC) gating was used to identify cells of interest based on size and granularity and exclude debris. Then a forward scatter area (FSC-A) vs forward scatter height (FSC-H) density plot was used to exclude doublets. At last, a two-parameter (PI, FITC) density blot was used and the phospho- histone 3 positive population (*i.e.* mitotic cells) were boxed. Related to Fig. **2g**.
