# Supplementary material for: A synergetic effect of BARD1 mutations on tumorigenesis
Source: Nat Commun. 2021 Feb 23;12:1243. doi: 10.1038/s41467-021-21519-3 (PMC7902612; doi:10.1038/s41467-021-21519-3)
Supplement: Supplementary file 2 — Description of Additional Supplementary Files [file 41467_2021_21519_MOESM2_ESM.pdf]

Description of Additional Supplementary Files

File Name: Supplementary Data 1
Description: Detail information of the hereditary breast and ovarian cancer family.

File Name: Supplementary Data 2
Description: General WGS information.

File Name: Supplementary Data 3
Description: Potential pathogenic SNPs.

File Name: Supplementary Data 4
Description: Potential pathogenic INDELs.

File Name: Supplementary Data 5
Description: Primer name and sequence.

File Name: Supplementary Data 6
Description: Linkdat format data of the pedigree.
