# Supplementary material for: A synergetic effect of BARD1 mutations on tumorigenesis
Source: Nat Commun. 2021 Feb 23;12:1243. doi: 10.1038/s41467-021-21519-3 (PMC7902612; doi:10.1038/s41467-021-21519-3)
Supplement: Supplementary file 9 — Reporting Summary [file 41467_2021_21519_MOESM9_ESM.pdf]

nature research

Corresponding author(s): Mo Li

Last updated by author(s): Dec 31, 2020

Reporting Summary

Nature Research wishes to improve the reproducibility of the work that we publish. This form provides structure for consistency and transparency in reporting. For further information on Nature Research policies, see our Editorial Policies and the Editorial Policy Checklist.

Statistics

For all statistical analyses, confirm that the following items are present in the figure legend, table legend, main text, or Methods section.

n/a	Confirmed	
☐	☒	The exact sample size (*n*) for each experimental group/condition, given as a discrete number and unit of measurement
☐	☒	A statement on whether measurements were taken from distinct samples or whether the same sample was measured repeatedly
☐	☒	The statistical test(s) used AND whether they are one- or two-sided<br>*Only common tests should be described solely by name; describe more complex techniques in the Methods section.*
☐	☒	A description of all covariates tested
☐	☒	A description of any assumptions or corrections, such as tests of normality and adjustment for multiple comparisons
☐	☒	A full description of the statistical parameters including central tendency (e.g. means) or other basic estimates (e.g. regression coefficient) AND variation (e.g. standard deviation) or associated estimates of uncertainty (e.g. confidence intervals)
☐	☒	For null hypothesis testing, the test statistic (e.g. *F*, *t*, *r*) with confidence intervals, effect sizes, degrees of freedom and *P* value noted<br>*Give P values as exact values whenever suitable.*
☒	☐	For Bayesian analysis, information on the choice of priors and Markov chain Monte Carlo settings
☐	☒	For hierarchical and complex designs, identification of the appropriate level for tests and full reporting of outcomes
☐	☒	Estimates of effect sizes (e.g. Cohen's *d*, Pearson's *r*), indicating how they were calculated

Our web collection on statistics for biologists contains articles on many of the points above.

Software and code

Policy information about availability of computer code

Data collection	bcl2fastq2 Conversion Software (version 2.20)<br>cellSens Dimension (version 2.3)<br>ZEN (version 2.0 black) for Carl Zeiss 710<br>ZEN (version 2.3 black) for Carl Zeiss 880<br>Volocity (version 6.4.0)<br>Living Image Software (version 3.10)<br>NTControl Software (version 2.2.1)<br>GelCap ECL (version 5.6)<br>Image Lab (version 4.1)
Data analysis	1.WGS Data Processing:<br>BWA (version 0.7.17)<br>SAMtools (version 1.10)<br>Picard (version 2.21.9)<br>GATK (version 4.1.7.0)<br>ANNOVAR (version 2018Apr16)<br><br>2.Image Analysis:<br>ZEN (version 2.0 blue)<br>Comet Assay software (version IV)<br>Living Image software (version 3.10)<br>ImageJ (version 1.51)<br><br>3.Others:

Paramlink (version 1.1-2, https://CRAN.R-project.org/package=paramlink)
CNV-seq (http://tiger.dbs.nus.edu.sg/cnv-seq/)
FlowJo Software (version 10.5.3)
MO. Affinity Analysis (version 2.1.3)
Gromacs 2018.6

4.Statistical Analyses:
GraphPad Prism (version 7.00)

For manuscripts utilizing custom algorithms or software that are central to the research but not yet described in published literature, software must be made available to editors and reviewers. We strongly encourage code deposition in a community repository (e.g. GitHub). See the Nature Research guidelines for submitting code & software for further information.

Data

Policy information about availability of data

All manuscripts must include a data availability statement. This statement should provide the following information, where applicable:
- Accession codes, unique identifiers, or web links for publicly available datasets
- A list of figures that have associated raw data
- A description of any restrictions on data availability

The WGS data reported in this study have been deposited in the genome sequence archive of Beijing Institute of Genomics, Chinese Academy of Sciences, Beijing, China (gsa.big.ac.cn, accession no. CRA002326). The solution NMR structure of the BRCA1/BARD1 RING-domain heterodimer is available from http://www1.rcsb.org/structure/1JM7. The remaining data are available within the Article, Supplementary information or available from the authors upon request.

Field-specific reporting

Please select the one below that is the best fit for your research. If you are not sure, read the appropriate sections before making your selection.

☒ Life sciences ☐ Behavioural & social sciences ☐ Ecological, evolutionary & environmental sciences

For a reference copy of the document with all sections, see nature.com/documents/nr-reporting-summary-flat.pdf

Life sciences study design

All studies must disclose on these points even when the disclosure is negative.

Sample size	No statistical methods were used to predetermine sample sizes. The sample sizes were chosen according to previous experience and on what is common practice in the field. For WGS, the number of sequenced samples of involved participants was determined by the proband and the related members in the family of Hereditary Breast and Ovarian Cancer.
Data exclusions	No data were excluded from the analyses.
Replication	The experiments were performed in triplicate unless otherwise stated. Replication information is included in the Figure Legends and/or text. All replicates are sufficient for us to perform statistical tests when needed. All replicates were reproducible.
Randomization	Samples were random allocated into experimental groups.
Blinding	The investigators were blinded to group allocation during data collection and analysis.

Reporting for specific materials, systems and methods

We require information from authors about some types of materials, experimental systems and methods used in many studies. Here, indicate whether each material, system or method listed is relevant to your study. If you are not sure if a list item applies to your research, read the appropriate section before selecting a response.

Materials & experimental systems

n/a	Involved in the study	
☐	☒	Antibodies
☐	☒	Eukaryotic cell lines
☒	☐	Palaeontology and archaeology
☐	☒	Animals and other organisms
☐	☒	Human research participants
☒	☐	Clinical data
☒	☐	Dual use research of concern

Methods

n/a	Involved in the study	
☒	☐	ChIP-seq
☐	☒	Flow cytometry
☒	☐	MRI-based neuroimaging

Antibodies

Antibodies used

Rabbit polyclonal anti-BARD1 antibody (Abcam, Cat#ab226854), IF: 1:100 dilution
Rabbit monoclonal anti-estrogen receptor antibody (Abcam, Cat#ab16660), IHC: 1:200 dilution
Mouse monoclonal anti-β-actin antibody (Abcam, Cat#ab8226), WB: 1:2000 dilution
Rabbit monoclonal anti-progesterone receptor antibody (Cell Signaling Technology, Cat#8757), IHC: 1:1000 dilution
Rabbit monoclonal anti-HER2 antibody (Cell Signaling Technology, Cat#2165), IHC: 1:200 dilution
Rabbit monoclonal anti-TOPOIIα antibody (Cell Signaling Technology, Cat#12286s), WB: 1:1000 dilution
Rabbit monoclonal anti-GFP antibody (Cell Signaling Technology, Cat#2956), WB: 1:1000 dilution
Rabbit monoclonal anti-γH2AX antibody (Cell Signaling Technology, Cat#2577S), IF: 1:100 dilution
Rabbit monoclonal anti-phospho-histone H3 (Ser10) antibody (Cell Signaling Technology, Cat#9701s), Flow Cyt: 1:50 dilution
Mouse monoclonal anti-BRCA1 antibody (Santa, Cat#sc-6954), WB: 1:200; IF: 1:50 dilution
Mouse monoclonal anti-P53 antibody (Novus, Cat#NB200-103), IHC: 1:250; WB: 1:1000 dilution
Alexa FluorTM 633 goat anti-mouse IgG (Thermo, Cat#A-21126), IF: 1:200 dilution
Alexa FluorTM 555 goat anti-Rabbit IgG (Thermo, Cat#A-21429), IF: 1:200 dilution
HRP goat anti-mouse IgG (H+L) secondary antibody (Thermo, Cat#32430), WB: 1:1000 dilution
HRP goat anti-rabbit IgG(H+L) secondary antibody (Thermo, Cat#31466), WB: 1:1000 dilution

Validation

Anti-BARD1 antibody (ab226854; Suitable for: WB, IHC-P, ICC/IF; Reacts with: Mouse, Rat, Human; https://www.abcam.com/bard1-antibody-ab226854.html)
Anti-estrogen receptor antibody (ab16660; Suitable for: WB, IHC-P, ICC/IF, Flow Cyt; Reacts with: Human; https://www.abcam.com/estrogen-receptor-alpha-antibody-sp1-ab16660.html)
Anti-β-actin antibody (ab8226; Suitable for: ICC/IF, IHC-P, WB; Reacts with: Mouse, Rat, Rabbit, Chicken, Cow, Dog, Human, Pig, African green monkey, Chinese hamster, Armenian hamster; https://www.abcam.cn/beta-actin-antibody-mabcam-8226-loading-control-ab8226.html)
Anti-progesterone receptor antibody (8757; Suitable for: WB, IHC-P, ICC/IF, Flow Cyt; Reacts with: Human; https://www.cellsignal.com/products/primary-antibodies/progesterone-receptor-a-b-d8q2j-xp-rabbit-mab/8757)
Anti-HER2 antibody (2165; Suitable for: WB, IHC-P, ICC/IF, Flow Cyt; Reacts with: Human, Mouse; https://www.cellsignal.com/products/primary-antibodies/her2-erbb2-29d8-rabbit-mab/2165)
Anti-TOPOIIα antibody (12286s; Suitable for: WB, IHC-P, ICC/IF, Flow Cyt; Reacts with: Human; https://www.cellsignal.cn/products/primary-antibodies/topoisomerase-iia-d10g9-xp-rabbit-mab/12286)
Anti-GFP antibody (2956; Suitable for: WB, IHC-P; Reacts with: All; https://www.cellsignal.com/products/primary-antibodies/gfp-d5-1-xp-rabbit-mab/2956)
Anti-γH2AX antibody (2577S; Suitable for: WB, ICC/IF, Flow Cyt; Reacts with: Human, Mouse; https://www.cellsignal.com/products/primary-antibodies/phospho-histone-h2a-x-ser139-antibody/2577)
Anti-phospho-histone H3 (Ser10) antibody (9701s; Suitable for: WB, IHC-P, ICC/IF, Flow Cyt; Reacts with: Human, Mouse; https://www.cellsignal.cn/products/primary-antibodies/phospho-histone-h3-ser10-antibody/9701)
Anti-BRCA1 antibody (sc-6954; Suitable for: WB, ICC/IF; Reacts with: Human; https://www.scbt.com/p/brca1-antibody-d-9)
Anti-P53 antibody (NB200-103; Suitable for: WB, IHC-P, ICC/IF, Flow Cyt; Reacts with: Human, Mouse; https://www.novusbio.com/products/p53-antibody-pab-240_nb200-103)

Eukaryotic cell lines

Policy information about cell lines

Cell line source(s)

U2OS, MCF7 and MCF10A cells were obtained from ATCC.

Authentication

Because no commonly misidentified cell lines were used in the study, morphology check by microscope was done for each cell line for authentication.

Mycoplasma contamination

All cell lines were tested negative for mycoplasma contamination.

Commonly misidentified lines
(See ICLAC register)

No commonly misidentified cell lines were used in the study.

Animals and other organisms

Policy information about studies involving animals; ARRIVE guidelines recommended for reporting animal research

Laboratory animals

Female BALB/c nude mice aged 4-6 weeks were used.

Wild animals

The study did not involve wild animals.

Field-collected samples

The study did not involve samples collected from the field.

Ethics oversight

The Ethics Committee of Peking University Health Science Center approved the study protocol.

Note that full information on the approval of the study protocol must also be provided in the manuscript.

Human research participants

Policy information about studies involving human research participants

Population characteristics	The gender, age, cancer type of participants in the generation of proband (#11, 12, 15, 17, 19) are female/55/breast cancer, female/49/no cancer, female/41/no cancer, female/52/no cancer, and female/42/ovarian cancer, respectively.
Recruitment	The family were recruited in this study on account of a typical characteristics of HBOC family in which multiple cases of breast cancer and/or ovarian cancer occurred on the same side of the family. We sampled the peripheral blood of all surviving female members including Person-11, -12, -15, -17, and -19 for whole genomic sequencing, so there is no self-selection bias that could impact results.
Ethics oversight	All human materials used in this study was approved by Peking University Third Hospital Medical Science Research Ethic Committee (IRB00006761-M2019343). Signed informed consents were obtained from the family members who participated in the study.

Note that full information on the approval of the study protocol must also be provided in the manuscript.

Flow Cytometry

Plots

Confirm that:

☒ The axis labels state the marker and fluorochrome used (e.g. CD4-FITC).

☒ The axis scales are clearly visible. Include numbers along axes only for bottom left plot of group (a 'group' is an analysis of identical markers).

☒ All plots are contour plots with outliers or pseudocolor plots.

☒ A numerical value for number of cells or percentage (with statistics) is provided.

Methodology

Sample preparation	Cells expressing WT or mutant BARD1 variants were treated with or without 2 Gy of IR. After 1 hour of recovery, cells were fixed with 70% (v/v) ethanol, stained with rabbit antibody to phospho-histone H3 (pSer10), and then incubated with fluorescence-conjugated goat secondary antibody against rabbit. The stained cells were treated with RNase A and then dyed with propidium iodide.
Instrument	Flow Cytometer (BD FACSCelesta, BD Biosciences-US) was used for data collection.
Software	FlowJo V10 software was used to collect and analyze the flow cytometry data.
Cell population abundance	No post-sort fraction was used in the study.
Gating strategy	FSC/SSC gates were used to remove debris and doublets while preserving single living cells based on size and complexity. The boundaries between “positive” and “negative” cell populations were clear and the “positive” populations were circled.

☒ Tick this box to confirm that a figure exemplifying the gating strategy is provided in the Supplementary Information.
